# A pH/ROS Cascade‐Responsive Charge‐Reversal Nanosystem with Self‐Amplified Drug Release for Synergistic Oxidation‐Chemotherapy

**DOI:** 10.1002/advs.201801807

**Published:** 2018-12-18

**Authors:** Liangliang Dai, Xiang Li, Xianglong Duan, Menghuan Li, Peiyun Niu, Huiyun Xu, Kaiyong Cai, Hui Yang

**Affiliations:** ^1^ Institute of Medical Research Northwestern Polytechnical University Xi'an 710072 P. R. China; ^2^ School of Life Sciences Northwestern Polytechnical University Xi'an 710072 P. R. China; ^3^ Second Department of General Surgery Shaanxi Provincial People's Hospital Xi'an 710068 P. R. China; ^4^ Key Laboratory of Biorheological Science and Technology Ministry of Education College of Bioengineering Chongqing University Chongqing 400044 P. R. China

**Keywords:** cascade‐response, charge‐reversal, oxidation‐chemotherapy, prodrug micelles, self‐amplifiable drug release

## Abstract

Poor cell uptake of drugs is one of the major challenges for anticancer therapy. Moreover, the inability to release adequate drug at tumor sites and inherent multidrug resistance (MDR) may further limit the therapeutic effect. Herein, a delivery nanosystem with a charge‐reversal capability and self‐amplifiable drug release pattern is constructed by encapsulating β‐lapachone in pH/ROS cascade‐responsive polymeric prodrug micelle. The surface charge of this micellar system would be converted from negative to positive for enhanced tumor cell uptake in response to the weakly acidic tumor microenvironment. Subsequently, the cascade‐responsive micellar system could be dissociated in a reactive oxygen species (ROS)‐rich intracellular environment, resulting in cytoplasmic release of β‐lapachone and camptothecin (CPT). Furthermore, the released β‐lapachone is capable of producing ROS under the catalysis of nicotinamide adenine dinucleotide (NAD)(P)H:quinone oxidoreductase‐1 (NQO1), which induces the self‐amplifiable disassembly of the micelles and drug release to consume adenosine triphosphate (ATP) and downregulate P‐glycoprotein (P‐gp), eventually overcoming MDR. Moreover, the excessive ROS produced from β‐lapachone could synergize with CPT and further propagate tumor cell apoptosis. The studies in vitro and in vivo consistently demonstrate that the combination of the pH‐responsive charge‐reversal, upregulation of tumoral ROS level, and self‐amplifying ROS‐responsive drug release achieves potent antitumor efficacy via the synergistic oxidation‐chemotherapy.

## 1 Introduction

Combining the advantages of polymer micelles (e.g., good bionic characteristics) and prodrug strategy (e.g., high drug loading capacity), polymeric prodrug micellar‐based drug delivery systems (PPM‐DDS) were extensively explored to overcome the drawbacks of conventional chemotherapy (e.g., poor tumor selectivity and severe side effects) and improve the antitumor efficacy. Thus, PPM‐DDS has emerged as a promising platform for tumor therapy.^1^, ^2^ Taking the advantage of prodrug and stimuli‐responsive drug release behavior, PPM‐DDS could remarkably increase the targeting efficiency against tumors through the enhanced permeability and retention effect (EPR), therefore improving the bioavailability and therapeutic effects of anticancer drugs.^3^, ^4^

The poor tumor cells' uptake and incomplete drug release are the two critical challenges hindering the clinical translation of PPM‐DDS.^5^ To improve the therapeutic efficacy of PPM‐DDS, tremendous efforts have been devoted to the development of stimuli‐responsive tumor‐targeted drug delivery systems.^6^ On one hand, PPM‐DDS would maintain their stealth features during circulation and then undergo a transformation process once exposed to tumor microenvironment for strong cellular binding to achieve enhanced tumor cells' internalization.^7^ Typically, pH‐dependent charge conversion strategy was utilized for the construction of PPM‐DDS for tumor‐targeted drug delivery, since tumor having weakly acidic microenvironment (pH ≈ 6.8).^8^, ^9^ The pH‐dependent charge reversal delivery systems could remain negatively charged under physiological environment (pH 7.4) to reduce nonspecific interactions with serum components and avoid clearance by reticuloendothelial system (RES),^10^ while they could be converted to positive upon weakly acidic pH to enhance targeted tumor uptake.^11^ On the other hand, after cells endocytosis, the PPM‐DDS should possess ultrasensitive drug release property in a tumor‐specific manner as well.^12^ It is found that the concentration of reactive oxygen species (ROS), including hydrogen peroxides (H_2_O_2_), hydroxyl radicals (OH·), and superoxides (O_2_^−^), in tumor cells is significantly higher than that of normal cells;^13^ thus, PPM‐DDS with ROS‐responsive drug release feature is a powerful strategy to achieve the selective drug release in tumor cells. Various ROS‐responsive copolymers comprising of oxidation‐labile groups such as thioketal, alkylene sulfide, and boronic ester have been extensively investigated to construct DDS for tumor therapy.^14^ Nevertheless, the aforementioned ROS‐responsive mechanism may be affected by the tumor heterogeneity, where the endogenous ROS concentration is not high enough to activate the complete drug release.^15^, ^16^ Therefore, pH/ROS‐responsive charge‐reversal PPM‐DDS with ROS generation capability is a promising alternative that could significantly increase drug release selectivity and tumor therapeutic efficacy.

Compared to typical photosensitizers, β‐lapachone exhibits superior tumor‐activating ROS generation ability. The β‐lapachone could be catalyzed by NAD(P)H:quinone oxidoreductase‐1 (NQO1) enzyme that is overexpressed 100‐fold in tumor cells than normal cells,^17^ and its implementation is not limited by the laser penetration depth. Meanwhile, the generation of ROS by β‐lapachone is accompanied with the consumption of NAD(P)H/ATP and downregulation of P‐glycoprotein (P‐gp).^13^, ^18^ It thus would suppress adenosine triphosphate (ATP)‐dependent drug efflux mediating by P‐gp, improve the bioavailability of chemotherapeutic drugs, and overcome multidrug resistance (MDR). Therefore, to load β‐lapachone into ROS‐responsive drug delivery systems would remarkably amplify oxidative stress and reduce MDR for complete drug release and synergistic oxidation therapy.

**Scheme 1:**
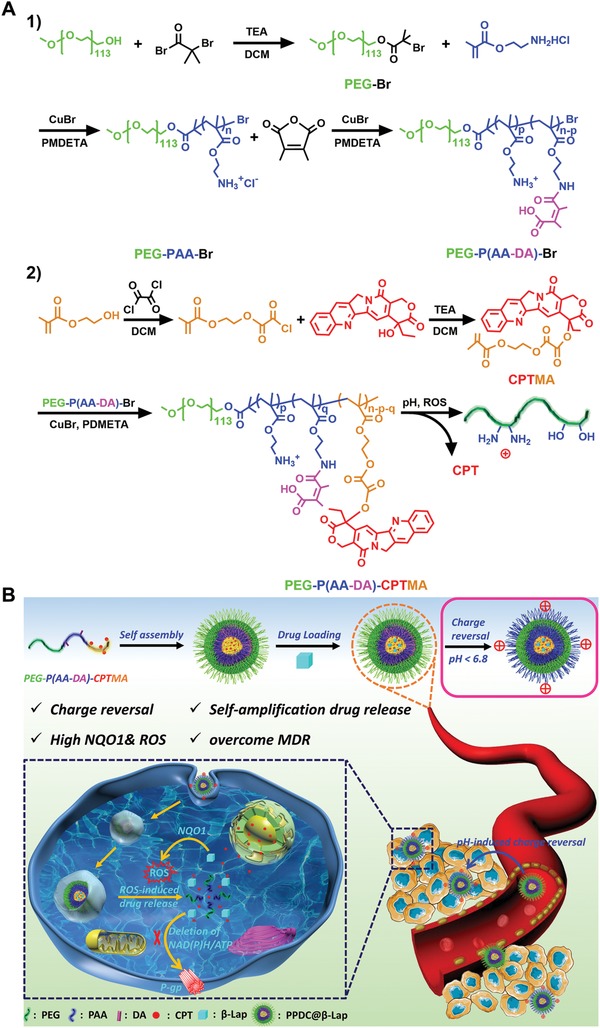
A) Synthesis routes and disassembly mechanism of pH/ROS cascade‐responsive prodrug copolymer PEG—P(AA—DA)—CPTMA. B) Illustration of the charge reversal PPDC system with self‐amplifiable drug release for tumor therapy in vivo.

To address the aforementioned concerns, herein, we report a self‐amplifiable drug release system with charge reversal ability by loading β‐lapachone in a pH/ROS cascade‐responsive polymeric prodrug micelle polyethylene glycol (PEG)—P(2‐aminoethyl methacrylate hydrochloride (AA)—DA)—camptothecin conjugated hydroxyethyl methacrylate‐oxalyl chloride (CPTMA) (denoted as PPDC@β‐Lap). PEG—P(AA—DA)—CPTMA was synthesized via sequential atom transfer radical polymerization (ATRP) with PEG as the hydrophilic outer layer. A moderate amount of dimethylmaleic anhydride (DA) was conjugated to the backbone of copolymer as the middle layer, endowing the pH‐triggered charge reversal property. Hydrophobic camptothecin (CPT) prodrug linked by ROS‐responsive bonds was integrated into the copolymer as the inner core (**Scheme** **1** A). The system could offer several practical benefits in a programmed fashion (Scheme 1 B): i) the shielding PEG layer and negative surface charge of the micelles could prolong their circulation time and reduce the nonspecific clearance by RES;^19^ ii) the pH‐sensitive amides linked with DA would be hydrolyzed and shed the negatively charged DA molecules once exposing to the acidic pH in tumor microenvironment. It would result in the charge reversal from negative to positive and increase the tumor cells uptake; iii) after internalization, the endogenous ROS would induce the release of CPT from PPDC micelles by breaking the H_2_O_2_‐cleavable linkage. Moreover, the removal of CPT would disrupt the hydrophilic–hydrophobic balance of micelles and induce the disassembly of the micelles and drug release; iv) the released β‐lapachone could produce ROS and consume NAD(P)H/ATP, consequently amplifying the micelle disassembly and drug release and suppressing drug efflux. Finally, β‐lapachone could synergize with CPT and reverse MDR to aggravate tumor apoptosis. Thus, we hypothesize that PPDC@β‐Lap system could effectively enhance tumor therapeutic efficacy with reduced side effects via the synergistic oxidation‐chemotherapy.

## 2 Results and Discussion

### 2.1 Synthesis and Characterization of Micelles

**Figure 1:**
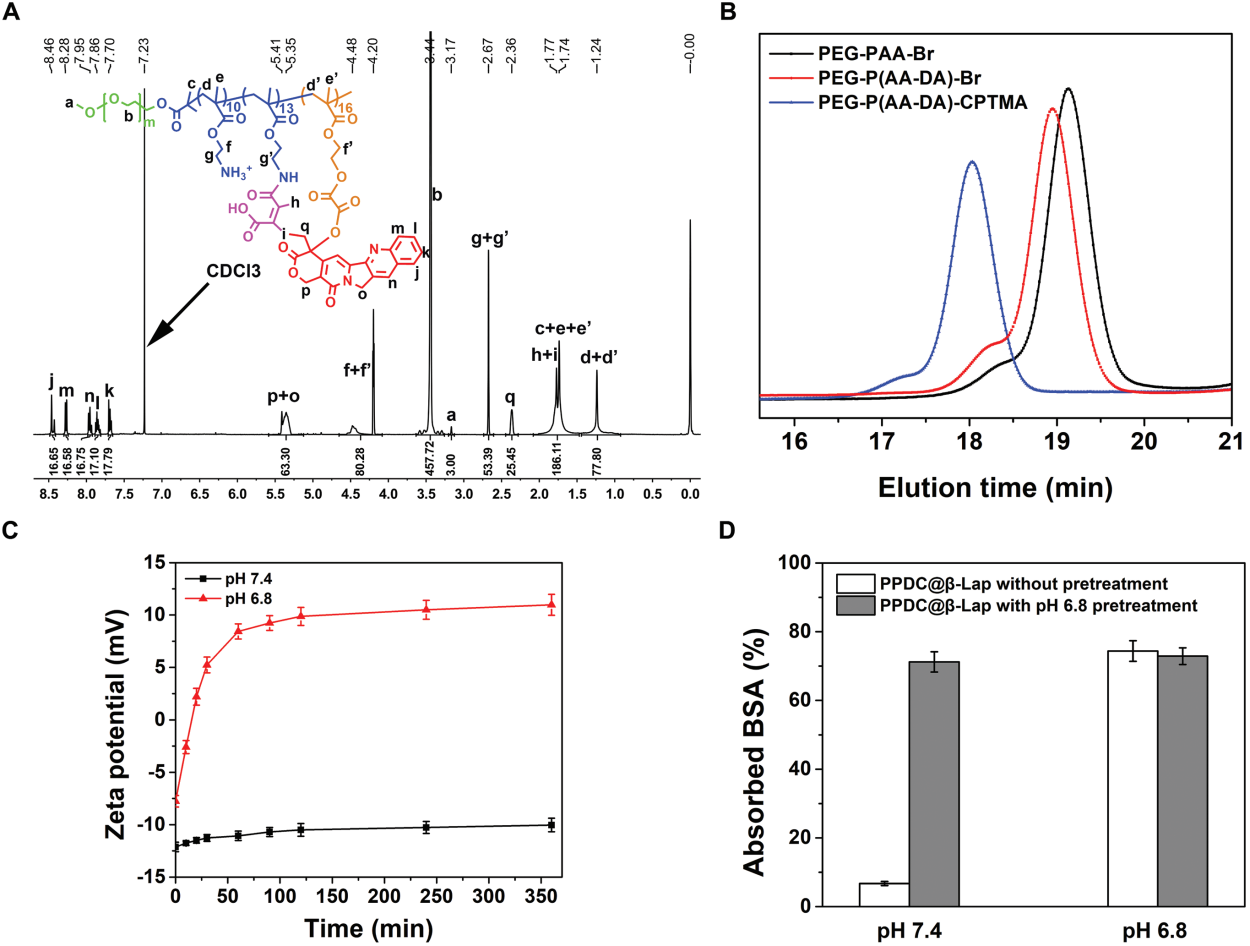
A) ^1^H NMR spectra of final copolymer PEG—P(AA—DA)—CPTMA. B) GPC traces (using tetrahydrofuran (THF) as the medium) of PEG—PAA—Br, PEG—P(AA—DA)—Br, and PEG—P(AA—DA)—CPTMA, respectively. C) Changes of zeta potentials of PPDC@β‐Lap micelles in phosphate buffered solution (PBS) at pH 7.4 and 6.8, respectively. D) BSA adsorption on the PPDC@β‐Lap micelles pretreated without/with pH 6.8 at different pH values.

The synthetic route of the pH/ROS cascade‐responsive prodrug polymer PEG—P(AA—DA)—CPTMA was illustrated in Scheme 1 A. Typically, PEG—P(AA—DA)—CPTMA was synthesized via ATRP copolymerization and stepwise chemical grafting reactions.^20^ The key components of the nanosystem comprise PEG, tumor acidity‐activated charge‐reversal layer, and ROS‐sensitive CPT prodrug segments, which was determined by ^1^H NMR spectroscopy and gel‐permeation chromatography (GPC) (**Figure** **1**A,B). Meanwhile, ^1^H NMR, mass spectrum, GPC, and Fourier transform infrared spectroscopy (FTIR) further validated the successful conjugations and chemical structure of PEG—P(AA—DA)—CPTMA copolymer (Figure 1B; Figures S1 and S2, Supporting Information). Besides, average molecular weights of various intermediate products calculated from ^1^H NMR and GPC were highly consistent with the theoretical values (Table S1, Supporting Information). The results further confirmed the successful synthesis of PEG—P(AA—DA)—CPTMA prodrug copolymer. The grafting ratio of CPT to the copolymer was calculated as 24.6%.

The empty micelles and β‐lapachone‐loaded micelles (denoted as PPDC and PPDC@β‐Lap) were prepared with the emulsion‐solvent evaporation method. Fluorescence study and stability assay indicated that the resulting PPDC micelles had a relatively low critical micelle concentration (CMC) of 1.2 µg mL^−1^ and good structural stability in 20% serum solution (Figures S3 and S4, Supporting Information). It would be helpful for overcoming the dilution effect in blood circulation and improving drug delivery.^2^, ^21^

### 2.2 Charge Reversal, ROS‐Responsive Micelles Disassembly and Drug Release

The two key features of the PPDC@β‐Lap system were the weakly acidic tumor microenvironment‐activated charge conversion and self‐amplifiable ROS‐responsive drug release. Surface potential of the micelles after incubation at pH 6.8 (simulation tumor microenvironment) was first monitored to investigate the pH‐dependent charge conversion. As shown in Figure 1C, PPDC@β‐Lap micelles remained strong negatively charge under physiological condition (pH 7.4) and only slightly changed after 4 h of incubation. The zeta potential of PPDC@β‐Lap micelles dramatically shifted from negative to positive (from −12.4 to +11.0 mV) at pH 6.8, which was caused by the gradual hydrolysis of DA in response to the weakly acidic pH.^22^ Notably, the removal of DA group had no influence on the size of micelles (Figure S5, Supporting Information). Bovine serum albumin (BSA) adsorption assay was further employed to confirm the pH‐responsive charge reversal process.^23^ As shown in Figure 1D, only a small quantity (below 7%) of BSA was adsorbed onto PPDC@β‐Lap micelles at pH 7.4 within 12 h, whereas, more than 72% of BSA adsorption was observed at pH 6.8 as the same duration. It was ascribed to the pH‐responsive charge conversion of PPDC system. Meanwhile, the PPDC@β‐Lap micelles incubated at pH 6.8 and pH 7.4 demonstrated high level of BSA adsorption after pretreatment at pH 6.8. It also confirmed the pH‐responsive charge reversal mechanism. It was noted that the PEG functionalized and negatively charged micelles could minimize the nonspecific serum protein absorption,^24^ which was consistent with the negligible size and polydispersity index (PDI) change observed after incubation in 20% serum for 6 days (Figure S4, Supporting Information). Moreover, the positively charged surface after charge reversion could enhance cell uptake and the following drug accumulation at tumor sites.^25^ Considering the weakly acidic tumor microenvironment, the pH‐responsive charge reversal of PPDC system could potentially enhance the drug delivery efficiency.

**Figure 2:**
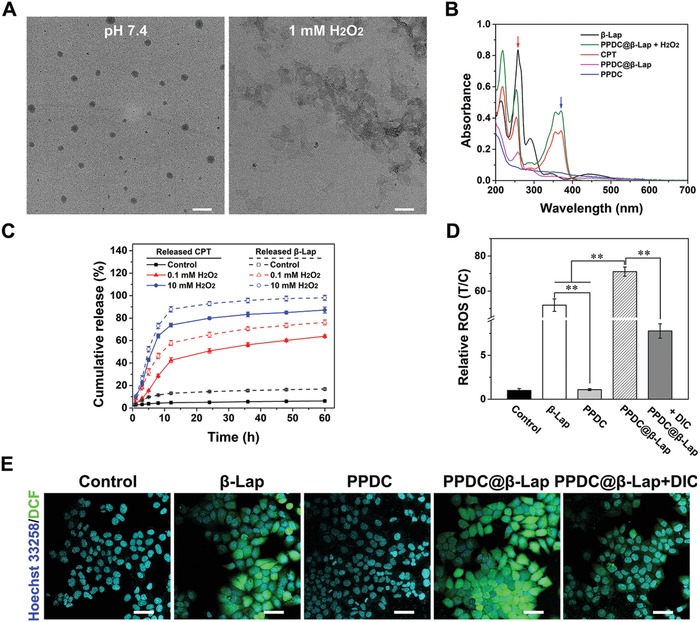
A) TEM images of PPDC@β‐Lap micelles in PBS (pH 7.4) without or with H_2_O_2_ for 2 h. Scale bars: 100 nm. B) The absorbance spectra of β‐lapachone, CPT, PPDC, and PPDC@@β‐Lap treated without or with 0.1 × 10^−3^ m H_2_O_2_ for 12 h in MeOH/H_2_O (1/4, v/v) solution. C) Cumulative release of CPT and β‐lapachone from PPDC@β‐Lap micelles after treatment with various concentrations of H_2_O_2_. D) FCM quantitative analysis and E) CLSM images of ROS levels in MCF‐7 cells treated with β‐lapachone, PPDC, and PPDC@β‐Lap without or with dicoumarol for 4 h. Nuclei and ROS were individually stained with Hoechst 333258 (blue) and DCFH‐DA (green). Scale bar: 50 µm. Results were presented as mean ± standard deviation (SD) (*n* = 6); **p < 0.01.

To investigate the sensitivity of micelles to ROS, transmission electron microscopy (TEM) and dynamic light scattering (DLS) were used to study the morphology and size change of PPDC@β‐Lap micelles in response to ROS stimuli. As shown in **Figure** **2**A, PPDC@β‐Lap exhibited a uniform spherical morphology with a hydrodynamic diameter of around 100 nm in the absence of H_2_O_2_ and was rapidly disintegrated upon the addition of H_2_O_2_. The morphological observation was consistent with the DLS results (Figure S6, Supporting Information), indicating the high sensitivity of the micellar system to ROS. The underlying mechanism is that the removal of CPT by ROS could transform the hydrophobic core to hydrophilic and then induce the disassembly of micelles. Considering the relatively low intracellular ROS concentration and the heterogeneity of tumors, β‐lapachone was also loaded into PPDC system to accelerate the drug release process. The absorbance spectra of PPDC@β‐Lap micelles showed a characteristic absorption peak of β‐lapachone (red arrow, Figure 2B), implying the successful encapsulation of β‐lapachone. The loading ratio and loading efficiency were calculated to be 16.8% and 71.2%, respectively. It was noted that both absorption intensity of encapsulated CPT and β‐lapachone were lower than their free forms. It was contributed to the successful drug loading of amphiphilic micelles and π–π stacking interactions.^13^, ^26^ In comparison, the characteristic absorbance of CPT and β‐lapachone dramatically increased after incubation with H_2_O_2_. It again confirms the ROS‐responsive micelle disassembly and drug release.

Subsequently, the quantitative analysis was employed to investigate the ROS‐responsive drug release behavior of micelles. As shown in Figure 2C, the control group exhibited negligible CPT and β‐lapachone leakage (both below 7%) within 60 h, indicating the good stability of the micelles. In contrast, around 63% CPT and 76% β‐lapachone, respectively, released from PPDC@β‐Lap micelles upon treatment with 0.1 × 10^−3^ m H_2_O_2_ (simulation intracellular ROS) for 60 h.^27^ Furthermore, when the H_2_O_2_ concentration increased to 10 × 10^−3^ m, the release percentage reached 87% for CPT and 96% for β‐lapachone. It again indicates the triggering effect of H_2_O_2_ concentration on drug release. These results consistently demonstrate the stability of the PPDC system under physiological condition and their ROS responses for controllable drug release.

### 2.3 Self‐Replenishment of ROS and self‐Amplifying Drug Release In Vitro

To study the self‐amplifying drug release of PPDC@β‐Lap micelles, we simultaneously examined the ROS generation efficiency by PPDC@β‐Lap in MCF‐7 cancer cells and its stimulating effect on drug release. In this study, we used dichlorofluorescein diacetate (DCF‐DA), an ROS indicator, which could be easily oxidized by ROS and produce dichlorofluorescein (DCF) with green fluorescence for staining.^2^, ^13^ MCF‐7 cells treated with β‐lapachone, PPDC, and PPDC@β‐Lap without or with dicoumarol (DIC, NQO1 inhibitor) were stained with DCFH‐DA kit, and the intracellular ROS generation was monitored using flow cytometer (FCM) and confocal laser scanning microscopy (CLSM), respectively. As shown in Figure 2D, both β‐lapachone and PPDC@β‐Lap rapidly produced ROS when comparing with the control and PPDC group (*p* < 0.01). Meanwhile, the treatment of PPDC@β‐Lap resulted in the highest ROS level, which was almost 80 times higher than that of control (Figure 2E). It indicates the potent ROS generation capability of PPDC@β‐Lap micelles. Moreover, ROS generation by micelles in MCF‐7 cells was evidently suppressed one adding the NQO1 inhibitor of DIC. It was suggested that the ROS generation by PPDC@β‐Lap system was dependent on NQO1. The PPDC@β‐Lap system thus possessed a tumor‐specific ROS generation and self‐amplifiable drug release behavior. It is especially relevant for tumor‐targeted drug delivery due to the overexpression of NQO1 in tumor cells.^13^, ^17^

### 2.4 Tumor‐Specific Cytotoxicity In Vitro

We subsequently examined the cytotoxicity of PPDC system against MCF‐7 cells using CCK‐8 assay. As shown in Figure S7A (Supporting Information), PPDC treatment induced similar level of cytotoxic damage to free CPT. The PPDC@β‐Lap group showed the lowest cell viability after the introduction of β‐lapachone in PPDC (*p* < 0.01). Furthermore, the NQO1 competitive inhibitor DIC remarkably decreased the cytotoxicity of PPDC@β‐Lap, which further validated that NQO1 was critical to the amplification of the drug release, since it could effectively catalyze the production of ROS in β‐lapachone‐loaded micelles (Figure 2D,E),^18^ resulting in the cascade‐amplified drug release and severe cytotoxicity. Additionally, nearly 80% of the normal NIH/3T3 cells with low NQO1 levels survived from treatment with the same concentration of PPDC@β‐Lap (Figure S7B, Supporting Information), implying the reduced cytotoxicity of the micellar system in normal cells. The results indicate that PPDCC@β‐Lap could effectively induce the tumor‐specific cytotoxicity and minimize the undesired side effect in vitro.

### 2.5 Enhanced Cell Uptake and Intracellular Drug Release of the Micelle System

**Figure 3:**
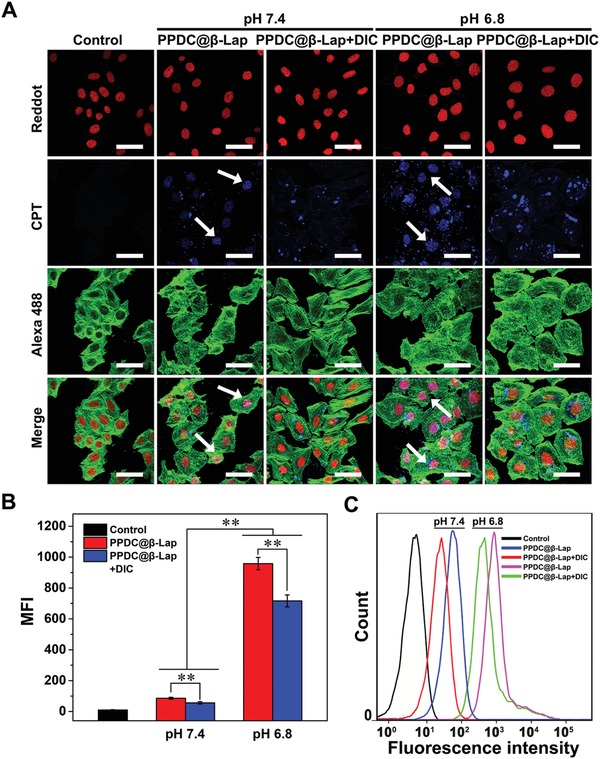
Cellular uptake and intracellular distribution of micelles: A) CLSM images, B) mean fluorescence intensity (MFI), and C) FCM analysis of CPT signal of PPDC@β‐Lap micelles (7.2 µg mL^−1^, equivalent of 5.1 × 10^−6^ m CPT) with or without the NQO1 inhibitor dicoumarol in MCF‐7 cells after incubation at pH 7.4 or 6.8 for 12 h, respectively. Nuclei and cytoskeleton were labeled with reddot2 (red) and Alexa 488‐phalloidin (green) individually. Scale bar: 50 µm. The collected data were presented as mean ± SD (*n* = 6); **p < 0.01.

To investigate the drug delivery efficiency of PPDC@β‐Lap system, the cell uptake and intracellular drug release in MCF‐7 cells under with various pH conditions or adding NQO1 inhibitor DIC were visualized and analyzed by CLSM and FCM. As demonstrated in **Figure** **3**A, upon culturing with PPDC@β‐Lap and PPDC@β‐Lap plus DIC at pH 6.8, cells exhibited much stronger CPT fluorescence compared to those at pH 7.4. A high amount of CPT has entered the nuclei (white arrow), revealing by the significant overlapping region between blue and red fluorescence. The result indicates the efficient endocytosis and micelle disassembly and drug release. Moreover, the amount of the endocytosed PPDC@β‐Lap in MCF‐7 cells evidently decreased upon co‐incubation with DIC regardless of pH values (Figure 3B, *p* < 0.01), which was revealed by the relatively weak blue fluorescence. These results were further supported by FCM analysis with similar trend (Figure 3C). It could be explained as follow: on one hand, the tumor acidity‐activating charge reversion enhanced cell adhesion on the negatively charged cell membranes and improved cell uptake of micelles.^28^ On the other hand, CPT and β‐lapachone would be released from micelles system in response to intracellular ROS. The released β‐lapachone generated more ROS via NQO1 catalysis (Figure 2D,E), which further accelerated and amplified the disassembly of micelles and drug release, thus leading to the highest CPT fluorescence intensity. Notably, DIC‐induced NQO1 inhibition could block the generation of ROS by β‐lapachone,^29^ resulting in impeded micelle disassembly and drug release. These results demonstrate that the tumor acidity‐activating surface charge‐reversible micelles with self‐amplifiable drug release were an promising platform with enhanced drug delivery efficiency.

### 2.6 PPDC@β‐Lap‐Induced MDR Inhibition, ATP Depletion, and cell Apoptosis In Vitro

**Figure 4:**
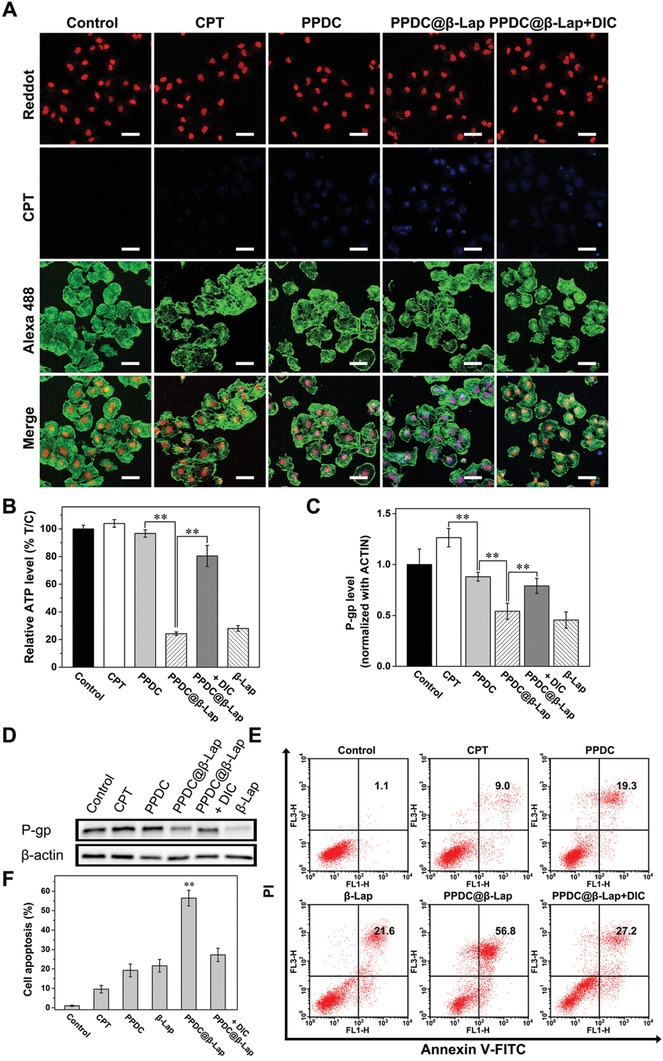
The potential mechanism of overcoming MDR. A) CLSM images of MCF‐7 ADR cells after treatment with CPT, PPDC, and PPDC@β‐Lap with or without the NQO1 inhibitor dicoumarol for 12 h, respectively. Nuclei and cytoskeleton were labeled with reddot2 (red) and Alexa 488‐phalloidin (green), respectively. Scale bar: 50 µm. B) Intracellular ATP level in MCF‐7 ADR cells treated with different formulations for 4 h. C) Quantitative analysis and D) western blotting images of P‐gp expression in MCF‐7 ADR cells after incubation different formulations for 48 h. β‐actin was used as control. E,F) Apoptosis analysis of MCF‐7 ADR cells induced by PBS (control), CPT (5.1 × 10^−6^ m), PPDC (7.2 µg mL^−1^, equivalent of 5.1 × 10^−6^ m CPT), and PPDC@β‐Lap (7.2 µg mL^−1^) without or with dicoumarol after 24 h of incubation determined using Annexin V‐FITC/PI staining and detected by FCM. Error bars present as mean ± SD (*n* = 6); **p < 0.01.

As NQO1‐mediated ROS generation of β‐lapachone is accompanied by ATP consumption, it would subsequently regulate expression of enzyme associated with drug efflux.^13^, ^30^ The ATP‐dependent P‐gp‐mediated drug efflux is critical to the development of MDR;^31^, ^32^ thus, PPDC@β‐Lap might provide extra benefit for overcoming MDR and enhancing antitumor efficiency. To verify this point, CLSM was used to assess MDR effect of micelles system against MCF‐7 multidrug resistant cells (ADR) drug‐resistant cells. As illustrated in **Figure** **4**A, CPT fluorescence intensity in MCF‐7 ADR cells was relatively low after treatment with CPT and PPDC for 12 h, owing to the native MDR. In contrast, cells treated by PPDC@β‐Lap displayed the highest CPT fluorescence intensity (blue), indicating that the PPDC@β‐Lap significantly suppressed MDR in MCF‐7 ADR cells. Moreover, the CPT fluorescence drastically decreased in MCF‐7 ADR cells after co‐treatment with PPDC@β‐Lap and DIC, implying that DIC strongly inhibited drug release, as also confirmed by the corresponding quantification analysis of CPT fluorescence intensity (Figure S8, Supporting Information). These results demonstrate that the NQO1‐catalyzed ROS generation by β‐lapachone played an important role in overcoming the MDR.

The above data demonstrated that PPDC@β‐Lap could enhance cellular uptake, decrease drug efflux mediated by MDR, and increase drug accumulation in MCF‐7 ADR cells. As a result, the high‐efficiency delivery of β‐Lapachone and CPT mediated by PPDC@β‐Lap system could significantly improve cytotoxicity and overcome MDR. To investigate the anti‐MDR effect of micelle system, we first incubated free CPT, PPDC, and PPDC@β‐Lap with MCF‐7 and examined its cytotoxicity with CCK‐8 assay. As shown in Figure S9 (Supporting Information), free CPT caused obvious cytotoxicity against MCF‐7 cells and weak cytotoxicity for MCF‐7 ADR cells with around 60% cell viability after incubation for 48 h. It was presumably owing to the native MDR‐mediated drug efflux. Meanwhile, PPDC@β‐Lap induced the most severe cytotoxicity against MCF‐7 cells (IC_50_ = 1.36 × 10^−6^ m), comparing with those of CPT (IC_50_ = 4.2 × 10^−6^ m) and PPDC (IC_50_ = 12.18 × 10^−6^ m). As for MCF‐7 ADR drug‐resistant cells, more importantly, the PPDC@β‐Lap displayed remarkably improved cytotoxicity as well, with around 12‐fold decrease in the IC_50_ value than that of CPT (6.56 × 10^−6^ m versus 74.27 × 10^−6^ m), indicating that MCF‐7 ADR cells were more sensitive to PPDC@β‐Lap than CPT and PPDC. The result again confirmed that PPDC@β‐Lap could indeed reverse MDR in cancer cells as reflected by the corresponding IC_50_ values.

To further clarify the MDR inhibition mechanism of PPDC@β‐Lap, we investigated the intracellular ATP levels in MCF‐7 ADR cells after treatment with different nanoformations. Only β‐lapachone and PPDC@β‐Lap groups exhibited the significantly reduction (around 75%, Figure 4B) of intracellular ATP level. The introduction of DIC remarkably suppressed the depletion of ATP level in MCF‐7 ADR cells. The ATP level increased to 85% of control group, again indicating that NQO1‐catalysized ROS generation by β‐lapachone was accompanied by ATP depletion. It thus would limit the ATP‐dependent drug efflux and improve the drug accumulation at tumor cells. In addition to ATP depletion, the PPDC@β‐Lap system also significantly downregulated the expression of MDR‐related P‐gp compared with control, CPT and PPDC groups (*p* < 0.01), as revealed by western blotting and corresponding quantitative analysis (Figure 4C,D). However, the expression of P‐gp in PPDC@β‐Lap + DIC group evidently increased, implying that NQO1‐catalyzed ROS generation by β‐lapachone was involved in the regulation of P‐gp expression through multiple pathways (e.g., nuclear factor‐κb (NF‐κB) and hypoxia‐inducible factor‐1α (HIF‐1α)). The results were consistent with previous studies.^13^, ^33^ The results confirmed that PPDC@β‐Lap could effectively reduce drug efflux and overcome MDR by blocking ATP supply and downregulating P‐gp expression, which was curial for enhanced tumor therapy.

To further demonstrate the enhanced antitumor effect of micelle system in vitro, FCM analysis was utilized to assess apoptosis level of MCF‐7 ADR cells after incubation with different formulations for 24 h. As shown in Figure 4E,F, compared to control, both CPT and β‐lapachone caused moderate apoptosis (9.0% and 21.6%), which was attributed to the chemotherapeutic and oxidative effects.^2^, ^34^ PPDC induced more severe apoptosis than that of the free CPT group, which was due to the superior drug delivery efficiency of charge reversible prodrug micelles.^35^ The PPDC@β‐Lap group induced the most severe apoptosis among all groups (56.8%), confirming the enhanced antitumor efficacy of oxidation‐chemotherapy of PPDC@β‐Lap system. Notably, the decreased apoptosis ratio in the PPDC@β‐Lap + DIC group was found to be associated with the less effective drug release, since DIC‐induced NQO1 inhibition could block the generation of ROS by Lap and thus limit the release of therapeutic agents. Additionally, the combination index (CI) calculated with the classic isobologram equation of Chou–Talalay was used to determine whether the effects were additive (CI = 1), synergistic (CI < 1), or antagonistic (CI > 1).^36^, ^37^ As shown in Table S2 (Supporting Information), the CI value of PPDC@β‐Lap was calculated as 0.44, which further confirmed their chemo‐photothermal synergetic therapeutic effects. These results reveal that the PPDC@β‐Lap could effectively induce cell apoptosis through the synergistic oxidation‐chemotherapy while simultaneously enhancing drug delivery efficacy and reducing MDR.

### 2.7 Antitumor Efficacy of the Combined Oxidation‐Chemotherapy In Vivo

**Figure 5:**
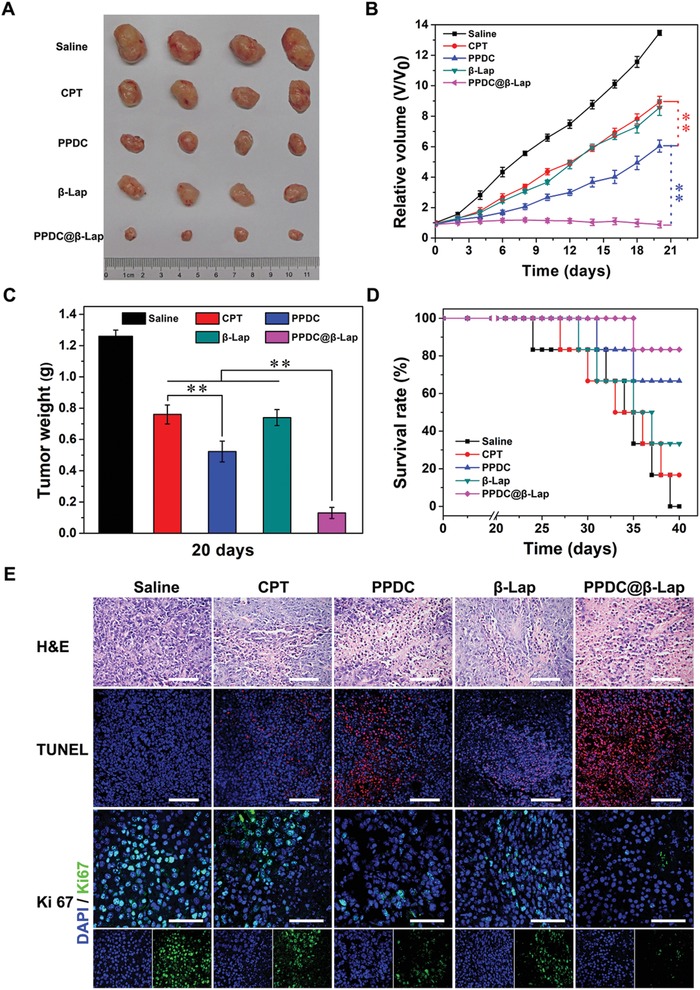
In vivo antitumor effects of micelles. A) Photographs of the tumors extracted from MDF‐7 tumor cell‐bearing mice after treatments with saline, CPT, β‐lapachone, PPDC and PPDC@β‐Lap (equivalent of 3 mg kg^−1^ CPT) for 20 days. B) Relative tumor volumes, C) final tumor weight, and D) survival rates of mice after various administrations. E) Images of H&E, TUNEL, and IFC with Ki67 staining of tumor sections, respectively. Scale bar: 100 µm. Error bars present as mean ± SD (*n* = 6), **p < 0.01.

To investigate the antitumor efficacy of PPDC@β‐Lap in vivo, MCF‐7 tumor‐bearing mouse model was constructed. As demonstrated in **Figure** **5**A, different treatments induced various extents of tumor growth suppression compared to control (saline). The chemotherapy of free CPT and oxidation therapy of β‐lapachone both caused moderate suppression of tumor growth. In comparison, the tumor growth inhibition of PPDC and PPDC@β‐Lap groups was higher than free CPT and β‐lapachone (*p* < 0.01), indicating the enhanced antitumor efficiency of the PPDC prodrug micelle system. More importantly, PPDC@β‐Lap induced the greatest tumor suppression among all groups without body weight loss (Figure S10, Supporting Information), and the tumor size was significantly reduced after treatment for 12 days (Figure 5B), indicating the superior antitumor effects. The above trend was further confirmed by tumor volume and final tumor weight analysis (Figure 5B,C). Additionally, PPDC@β‐Lap group also significantly prolonged the survival time of tumor‐bearing mice with a survive ratio of 83.3% after 40 days (Figure 5D), which was much higher than the other treatment groups, indicating again the superior antitumor efficiency. The reasons could be elucidated as follows: 1) the long‐circulating PEG layer and negative charged surface of PPDC@β‐Lap system could prolong the circulation time and increase nanocarrier accumulation at tumor sites through EPR effect;^38^ 2) tumor acidity‐activating charge conversion could effectively improve cell uptake of PPDC@β‐Lap;^39^ 3) after internalization, the endogenous ROS would induce micelle disassembly and drug release, and the released β‐lapachone could produce ROS for amplifying micelle disassembly and drug release; 4) β‐lapachone could synergize with CPT where it could reverse MDR and kill tumor cells with high efficiency. Above reasons contributed to the superior therapeutic efficacy of PPDC@β‐Lap system.

Hematoxylin‐eosin staining (H&E), terminal deoxynucleotidyl transferase‐mediated dUTP‐biotin nick end labeling assay (TUNEL), and immunofluorescence (IFC) staining assays were performed to further confirm the enhanced antitumor activity of the PPDC@β‐Lap system based on cell apoptosis. As shown in Figure 5E, PPDC and PPDC@β‐Lap groups induced more severe apoptosis than CPT and β‐lapachone, as revealed by the distinct cell shrinkage and chromatin condensation in H&E observation and abundant magenta dots co‐located with nuclei in TUNEL images.^40^ Meanwhile, PPDC@β‐Lap treatment induced the most severe cell apoptosis, which was consistent with IFC staining analysis of Ki67 for tumor tissues. PPDC@β‐Lap group displayed the lowest level of Ki67 expression among all groups, confirming the severe cell apoptosis.^41^ The results demonstrate that PPDC@β‐Lap system could effectively deliver CPT and β‐lapachone to tumor and induce tumor cells apoptosis/death with high efficiency in vivo.

### 2.8 In Vivo Biosafety Study

**Figure 6:**
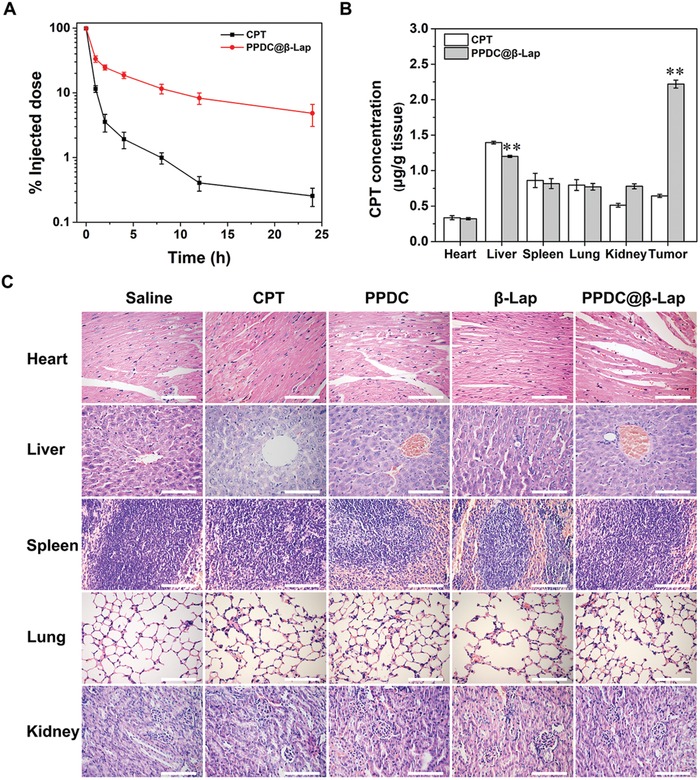
A) Pharmacokinetics of CPT and prodrug micelles PPDC@β‐Lap after intravenous injection into MCF‐7 cell tumor‐bearing mice at a CPT dose of 3 mg kg^−1^ for 24 h. B) Biodistribution of CPT in tumor‐bearing mice after intravenous injection of CPT and PPDC@β‐Lap for 24 h. C) Representative H&E images of the major organs (heart, spleen, lung, liver, and kidney) extracted from the mice after various treatments. Scale bar: 100 µm. Error bars present as mean ± SD (*n* = 4), **p < 0.01.

After confirming the superior antitumor effects, the biosafety of the PPDC@β‐Lap system in vivo was further investigated. We first monitored the in vivo pharmacokinetics of the PPDC@β‐Lap. As shown in **Figure** **6**A, compared with the relatively short blood circulation half‐life of CPT,^42^ PPDC@β‐Lap exhibited great circulation longevity under the same condition. It was attributed to the protection of PEG layer and negatively charged surface of the micelle system, which might also reduce nonspecific uptake clearance.^24^, ^43^ Considering the prolonged blood circulation would improve drug accumulation at tumor sites through EPR,^44^ we subsequently investigated the biodistribution of PPDC@β‐Lap system in vivo. It was observed that the greater amount of PPDC@β‐Lap accumulated in the tumor than that in other tissues. The amount of CPT delivered to tumor increased nearly five times compare to CPT (Figure 6B), suggesting that PPDC@β‐Lap could effectively deliver CPT to tumors via the EPR effect and pH‐activating charge reversal mechanism. Only a small amount of CPT was accumulated in tumor and the majority was presented in liver and lung, as confirmed by our previous work.^2^ It were caused by the poor solubility of the hydrophobic CPT, RES, and embolization of lung capillaries for hydrophobic CPT precipitation in the bloodstream,^45^ and eventually resulted in the rapid body weight loss (Figure S10, Supporting Information).

Finally, H&E staining was used to examine the morphological and histological changes of major tissues for further evaluating the biosafety of the PPDC@β‐Lap system. As confirmed by previous studies,^46^ CPT caused severe liver damage compared to the control owing to its side effect, indicating by the infiltration of inflammatory cells and decreasing hepatic cells (Figure 6C). In contrast, negligible liver damage was found in PPDC and PPDC@β‐Lap groups. Moreover, other organs in PPDC@β‐Lap treated mice also demonstrated the normal morphology (heart, kidneys, spleen, and lung), indicating the good biocompatibility of PPDC@β‐Lap micelles in vivo. The results consistently reveal that the PPDC@β‐Lap system significantly enhanced antitumor efficiency and reduced the side effects of CPT.

## 3 Conclusions

In summary, we developed a cascade‐responsive prodrug micelle drug delivery system with charge reversal and self‐amplifiable drug release for tumor therapy. The comprehensive in vitro and in vivo results revealed that the PPDC@β‐Lap not only effectively enhanced cell uptake and drug delivery via tumor acidity‐activating charge conversion and ROS‐response drug release, but also efficiently replenished the intratumoral ROS, reduced MDR via blocking APT supply, downregulated the expression of P‐gp to achieve complete drug release and suppressed drug efflux. It dramatically increased the tumor therapeutic efficacy with low systematic toxicity in vivo via synergistic oxidation‐chemotherapy. The work provides a promising drug release nanosystem with superior antitumor effect.

## Conflict of Interest

The authors declare no conflict of interest.

## Supporting information

Supplementary

## References

[1] D. Peer , J. M. Karp , S. Hong , O. C. Farokhzad , R. Margalit , R. Langer , Nat. Nanotechnol. 2007, 2, 751. 18654426 10.1038/nnano.2007.387

[2] L. L. Dai , R. S. Cai , M. H. Li , Z. Luo , Y. L. Yu , W. Z. Chen , X. K. Shen , Y. X. Pei , X. J. Zhao , K. Y. Cai , Chem. Mater. 2017, 29, 6976.

[3] J. Y. Liu , W. G. Liu , I. Weitzhandler , J. Bhattacharyya , X. H. Li , J. Wang , Y. Z. Qi , S. Bhattacharjee , A. Chilkoti , Angew. Chem., Int. Ed. 2015, 54, 1002. 10.1002/anie.201409293 PMC4293338 25427831

[4] M. E. Davis , Z. Chen , D. M. Shin , Nat. Rev. Drug Discovery 2008, 7, 771. 18758474 10.1038/nrd2614

[5] C. Deng , Y. J. Jiang , R. Cheng , F. H. Meng , Z. Y. Zhong , Nano Today 2012, 7, 467.

[6] E. Blanco , H. F. Shen , M. Ferrari , Nat. Biotechnol. 2015, 33, 941. 26348965 10.1038/nbt.3330 PMC4978509

[7] S. S. Han , Z. Y. Li , J. Y. Zhu , K. Han , Z. Y. Zeng , W. Hong , W. X. Li , H. Z. Jia , Y. Liu , R. X. Zhuo , X. Z. Zhang , Small 2015, 11, 2543. 25626995 10.1002/smll.201402865

[8] E. S. Lee , Z. Gao , D. Kim , K. Park , I. C. Kwon , Y. H. Bae , J. Controlled Release 2008, 129, 228. 10.1016/j.jconrel.2008.04.024 PMC2603624 18539355

[9] X. Guo , X. Wei , Y. T. Jing , S. B. Zhou , Adv. Mater. 2015, 27, 6450. 26401989 10.1002/adma.201502865

[10] X. Z. Yang , J. Z. Du , S. Dou , C. Q. Mao , H. Y. Long , J. Wang , ACS Nano 2012, 6, 771. 22136582 10.1021/nn204240b

[11] J. Z. Du , T. M. Sun , W. J. Song , J. Wu , J. Wang , Angew. Chem. 2010, 122, 3703.

[12] X. Guo , C. L. Shi , J. Wang , S. B. Di , S. B. Zhou , Biomaterials 2013, 34, 4544. 23510854 10.1016/j.biomaterials.2013.02.071

[13] M. Z. Ye , Y. X. Han , J. B. Tang , Y. Piao , X. R. Liu , Z. X. Zhou , J. Q. Gao , J. H. Rao , Y. Q. Shen , Adv. Mater. 2017, 29, 1702342.

[14] S. H. Lee , M. K. Gupta , J. B. Bang , H. Bae , H. J. Sung , Adv. Healthcare Mater. 2013, 2, 908. 10.1002/adhm.201200423 PMC4146500 25136729

[15] J. J. Li , W. D. Ke , L. Wang , M. M. Huang , W. Yin , P. Zhang , Q. X. Chen , Z. S. Ge , J. Controlled Release 2016, 225, 64. 10.1016/j.jconrel.2016.01.029 26806789

[16] L. L. Dai , Y. L. Yu , Z. Luo , M. H. Li , W. Z. Chen , X. K. Shen , F. Chen , Q. Sun , Q. F. Zhang , H. Gu , K. Y. Cai , Biomaterials 2016, 104, 1. 27423095 10.1016/j.biomaterials.2016.07.002

[17] X. P. Ma , X. M. Huang , Z. Moore , G. Huang , J. A. Kilgore , Y. G. Wang , S. Hammer , N. S. Williams , D. A. Boothman , J. M. Gao , J. Controlled Release 2015, 200, 201. 10.1016/j.jconrel.2014.12.027 PMC4803448 25542645

[18] E. Blanco , E. A. Bey , C. Khemtong , S. G. Yang , J. Setti‐Guthi , H. B. Chen , C. W. Kessinger , K. A. Carnevale , W. G. Bornmann , D. A. Boothman , J. M. Gao , Cancer Res. 2010, 70, 3896. 20460521 10.1158/0008-5472.CAN-09-3995 PMC2873165

[19] K. Xiao , Y. Li , J. Luo , J. S. Lee , W. Xiao , A. M. Gonik , R. G. Agarwal , K. S. Lam , Biomaterials 2011, 32, 3435. 21295849 10.1016/j.biomaterials.2011.01.021 PMC3055170

[20] P. D. Topham , N. Sandon , E. S. Read , J. Madsen , A. J. Ryan , S. P. Armes , Macromolecules 2008, 41, 9542.

[21] J. Lu , S. C. Owen , M. S. Shoichet , Macromolecules 2011, 44, 6002. 21818161 10.1021/ma200675w PMC3148800

[22] Q. Jiang , Y. Nie , X. B. Chen , Y. Y. He , D. Yue , Z. W. Gu , Adv. Funct. Mater. 2017, 27, 1701571.

[23] L. Jiang , L. Li , X. D. He , Q. Y. Yi , B. He , J. Cao , W. S. Pan , Z. W. Gu , Biomaterials 2015, 52, 126. 25818419 10.1016/j.biomaterials.2015.02.004

[24] J. J. Chen , J. X. Ding , Y. C. Yang , J. J. Cheng , S. X. Ji , X. L. Zhuang , X. S. Chen , Adv. Mater. 2017, 29, 1701170.

[25] L. L. Dai , K. Li , M. H. Li , X. J. Zhao , Z. Luo , L. Lu , Y. F. Luo , K. Y. Cai , Adv. Funct. Mater. 2018, 28, 1707249.

[26] Y. P. Li , T. Y. Lin , Y. Luo , Q. Q. Liu , W. W. Xiao , W. C. Guo , D. Lac , H. Y. Zhang , C. H. Feng , S. Wachsmann‐Hogiu , J. H. Walton , S. R. Cherry , D. J. Rowland , D. Kukis , C. X. Pan , K. S. Lam , Nat. Commun. 2014, 5, 4712. 25158161 10.1038/ncomms5712 PMC4145614

[27] C. De Gracia Lux , S. Joshi‐Barr , T. Nguyen , E. Mahmoud , E. Schopf , N. Fomina , A. Almutairi , J. Am. Chem. Soc. 2012, 134, 15758. 22946840 10.1021/ja303372u PMC3478073

[28] X. P. Duan , Y. P. Li , Small 2013, 9, 1521. 23019091 10.1002/smll.201201390

[29] A. Lewis , M. Ough , L. Li , M. M. Hinkhouse , J. M. Rithie , D. R. Spitz , J. J. Cullen , Clin. Cancer Res. 2004, 10, 4550. 15240547 10.1158/1078-0432.CCR-03-0667

[30] J. E. Lewis , F. Costantini , J. Mims , X. F. Chen , C. N. Furdui , D. A. Boothman , M. L. Kemp , Antioxid. Redox Signaling 2018, 29, 937. 10.1089/ars.2017.7048 PMC6104251 28762750

[31] K. M. Tainton , M. J. Smyth , J. T. Jackson , J. E. Tanner , L. Cerruti , S. M. Jane , P. K. Darcy , R. W. Johnstone , Cell Death Differ. 2004, 11, 1028. 15131592 10.1038/sj.cdd.4401440

[32] A. H. Schinkel , J. W. Jonker , Adv. Drug Delivery Rev. 2012, 64, 138.

[33] B. T. Choi , J. H. Cheong , Y. H. Choi , Anti‐Cancer Drugs 2003, 14, 845. 14597880 10.1097/00001813-200311000-00011

[34] J. Fang , T. Seki , H. Maeda , Adv. Drug Delivery Rev. 2009, 61, 290. 10.1016/j.addr.2009.02.005 19249331

[35] J. Liu , Y. R. Huang , A. Kumar , A. Tan , S. B. Jin , A. Mozhi , X. J. Liang , Biotechnol. Adv. 2014, 32, 693. 24309541 10.1016/j.biotechadv.2013.11.009

[36] T. C. Chou , Cancer Res. 2010, 70, 440. 20068163 10.1158/0008-5472.CAN-09-1947

[37] Z. Yang , R. Cheng , C. Y. Zhao , N. Sun , H. Y. Luo , Y. Chen , Z. R. Liu , X. Li , J. Liu , Z. M. Tian , Theranostics 2018, 8, 4097. 30128039 10.7150/thno.26195 PMC6096383

[38] A. Albanese , P. S. Tang , W. C. W. Chan , Annu. Rev. Biomed. Eng. 2012, 14, 1. 22524388 10.1146/annurev-bioeng-071811-150124

[39] H. Y. Tian , Z. P. Guo , L. Lin , Z. X. Jiao , J. Chen , S. Q. Gao , X. J. Zhu , X. S. Chen , J. Controlled Release 2014, 174, 117. 10.1016/j.jconrel.2013.11.008 24240012

[40] X. W. Ding , Y. Liu , J. H. Li , Z. Luo , Y. H. Hou , Y. Hu , B. L. Zhang , J. J. Liu , J. Zhou , K. Y. Cai . ACS Appl. Mater. Interfaces 2014, 6, 7395. 24749476 10.1021/am500818m

[41] X. Wei , Y. Wang , X. Xiong , X. Guo , L. Zhang , X. B. Zhang , S. B. Zhou , Adv. Funct. Mater. 2016, 26, 8266.

[42] D. Schmid , G. E. Jarvis , F. Fay , D. M. Small , M. K. Greene , J. Majkut , S. Spence , K. M. McLaughlin , K. D. McCloskey , P. G. Johnston , A. Kissenpfennig , D. B. Longley , C. J. Scott , Cell Death Dis. 2014, 5, e1454. 25299779 10.1038/cddis.2014.413 PMC4649518

[43] D. Putnam , Nat. Mater. 2006, 5, 439. 16738681 10.1038/nmat1645

[44] V. Torchilin , Adv. Drug Delivery Rev. 2011, 63, 131. 10.1016/j.addr.2010.03.011 20304019

[45] M. Y. Peng , S. Y. Qin , H. Z. Jia , D. W. Zheng , L. Rong , X. Z. Zhang , Nano Res. 2016, 9, 663.

[46] O. M. Y. Koo , I. Rubinstein , H. Önyüksel , Pharm. Res. 2011, 28, 776. 21132352 10.1007/s11095-010-0330-4 PMC3789645

