# Correction to “[A pH/ROS Cascade‐Responsive Charge‐Reversal Nanosystem with Self‐Amplified Drug Release for Synergistic Oxidation‐Chemotherapy]”

**DOI:** 10.1002/advs.202522120

**Published:** 2025-12-05

[L.L. Dai, X. Li, X.L. Duan, M.H. Li, P.Y. Niu, H.Y. Xu, K.Y. Cai, H. Yang. A pH/ROS Cascade‐Responsive Charge‐Reversal Nanosystem with Self‐Amplified Drug Release for Synergistic Oxidation‐Chemotherapy. Adv. Sci. 2019; 6: 1801807]

https://doi.org/10.1002/advs.201801807

[In the originally published article, Figure 6c contained an error inadvertently. The H&E image of the liver treated by the PPDC group was mistakenly applied to the saline group. The corrected image is presented below.]


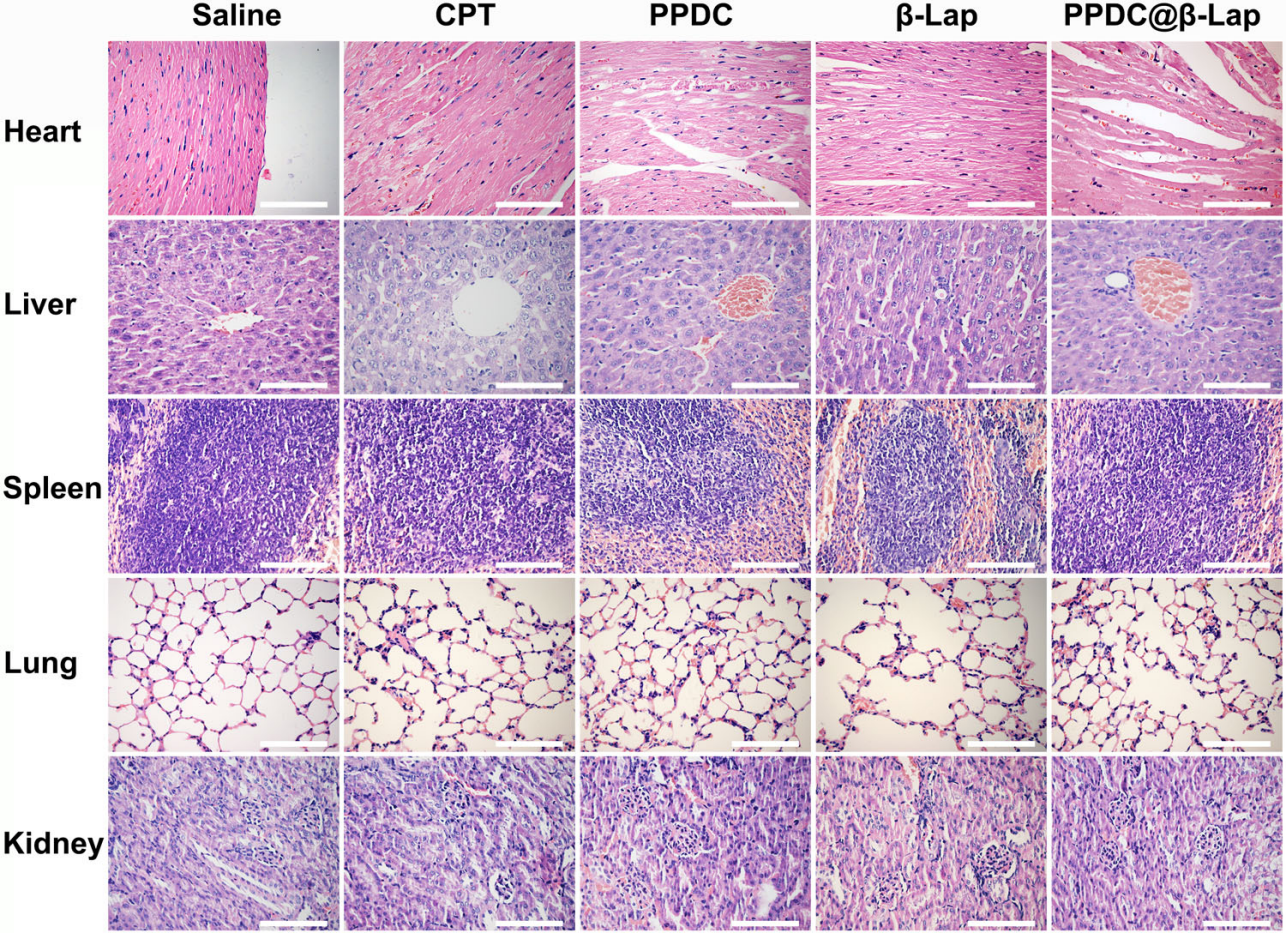


**Figure 6**. C) Representative H&E images of the major organs (heart, spleen, lung, liver, and kidney) extracted from the mice after various treatments.

We apologize for this error.

