# Supplementary material for: A pH/ROS Cascade‐Responsive Charge‐Reversal Nanosystem with Self‐Amplified Drug Release for Synergistic Oxidation‐Chemotherapy
Source: Adv Sci (Weinh). 2018 Dec 18;6(4):1801807. doi: 10.1002/advs.201801807 (PMC6382314; doi:10.1002/advs.201801807)
Supplement: Supplementary file 1 — Supplementary [file ADVS-6-1801807-s001.pdf]

Copyright WILEY-VCH Verlag GmbH & Co. KGaA, 69469 Weinheim, Germany, 2018.

ADVANCED SCIENCE
Open Access

Supporting Information

for *Adv. Sci.,* DOI: 10.1002/advs.201801807

A pH/ROS Cascade-Responsive Charge-Reversal Nanosystem with Self-Amplified Drug Release for Synergistic Oxidation-Chemotherapy

Liangliang Dai, Xiang Li, Xianglong Duan, Menghuan Li, Peiyun Niu, Huiyun Xu, Kaiyong Cai, and Hui Yang**

Copyright WILEY-VCH Verlag GmbH & Co. KGaA, 69469 Weinheim, Germany, 2016.

Supporting Information

PH/ROS Cascade-Responsive Charge-Reversal Nanosystem with Self-Amplified Drug Release for Synergistic Oxidation-Chemotherapy

Liangliang Dai, Xiang Li, Xianglong Duan, Menghuan Li, Peiyun Niu, Huiyun Xu, Kaiyong Cai, Hui Yang**

Dr. Liangliang Dai, Prof. Xianglong Duan, Prof. Hui Yang

Institute of Medical Research, Northwestern Polytechnical University, Xi'an 710072, P. R. China

E-mail: kittyyh@nwpu.edu.cn

Xiang Li, Peiyun Niu, Prof. Huiyun Xu, Prof. Hui Yang

School of Life Sciences, Northwestern Polytechnical University, Xi'an 710072, P. R. China

Prof. Xianglong Duan

Second Department of General Surgery, Shaanxi Provincial People's Hospital, Xi'an 710068, P. R. China

Dr. Menghuan Li, Prof. Kaiyong Cai

Key Laboratory of Biorheological Science and Technology, Ministry of Education, College of Bioengineering, Chongqing University, Chongqing 400044, P. R. China

E-mail: kaiyong_cai@cqu.edu.cn

List of Contents

Experimental Section……………………………………………………………………………………S3

Figure S1. NMR and ESI-MS spectra of the synthesis of PEG-P(AA-DA)-CPTMA prodrug copolymer……………………………………………………………………………………………S13

Figure S2. FTIR spectra of the synthesis of PEG-P(AA-DA)-CPTMA copolymer……………………S17

Figure S3. CMC characterization of micelles………………………………………………………………S18

Figure S4. The stability of micelles in serum……………………………………………………S19

Figure S5. TEM and particle size distribution (DLS) of micelles in pH 7.4 and pH 6.8…………S20

Figure S6. DLS of micelles in PBS (pH 7.4) without or with H2O2………………………………S21

Figure S7. Cell viability assay………………………………………………………………S22

Figure S8. Quantitative analysis of CPT fluorescence uptake in MCF-7 ADR cells with CLSM..S23

Figure S9. Cytotoxicity assays of PPDC@β-Lap in MCF-7 cells and MCF-7 ADR cells……………S24

Figure S10. The curve of weight change of mice……………………………………………S25

Table S1. Molecular weight parameters of copolymer…………………………………………S26

Table S2. IC50 and CI of different drug formulations..S27

References………S28

Experimental Section

Materials. 2-aminoethyl methacrylate hydrochloride, methoxy poly (ethylene glycol) (mPEG-OH, Mn 5000 g/mol), 2-bromoisobutyryl bromide, N,N-Dimethylacetamide, dimethylmaleic anhydride, 2-Hydroxyethyl methacrylate, 9, 10-anthracenediylbis (methylene) dimalonic acid (ABDA) and N,N,N',N'',N''-Pentamethyldiethylenetriamine were purchased from Sigma-Aldrich (Beijing, China). Copper(I) bromide (CuBr) and oxalic dichloride were purchased from Alfa Aesar (Tianjin, China). β-lapachone, triethylamine (TEA) and dichloromethane (DCM) were purchased from J&K Scientific Ltd. Dimethyl sulfoxide (DMSO) and sodium hydroxide were supplied by Aladdin Industrial Co., Ltd. Diethyl ether, ethanol and ethyl acetate were bought from Chuandong Chemical Co Ltd. (Chongqing, China). Primary antibodies including mouse anti-P-gp and anti-β-actin were obtained from Abcam (Cambridge, MA, USA) and Proteintech Group, Inc. (Wuhan, China), respectively. Secondary antibodies of horseradish peroxidase-conjugated goat anti-mouse IgG were purchased from Boster Biological Technology Co., Ltd (Wuhan, China). ActinGreen™ 488 ReadyProbes™ Reagent were bought from ThermoFisher Scientific (Waltham, MA, USA).

Synthesis of PEG-Br initiator. Typically, PEG113-OH (2 mmol) was dissolved in 10 mL of anhydrous dichloromethane (DCM) followed by the addition of anhydrous triethylamine (275 µL, 2 mmol). 2-bromoisobutyryl bromide (250 µL, 2 mmol) in anhydrous DCM was added dropwise into the mixture solution over 2 h in ice bath. The reaction would continue overnight at room temperature. The crude product was purified by diethyl ether precipitation and ethanol recrystallization (yield 80%). ^{1}H NMR (500 MHz, CDCl3): δ 1.94 (s, 6H, BrC(CH3)2), 3.38 (s, 3H, -OCH3), 3.64 (m, 454H, -OCH2CH2-). FTIR: 2887, 1111 cm^{-1}. The ^{1}H NMR and FTIR spectrum of PEG-Br were shown in **Figure S1** a and **Figure S2** a, respectively.

Synthesis of PEG-PAA-Br. PEG-PAA-Br was prepared via the sequential atom transfer radical polymerization (ATRP) using PEG-Br as the initiator. [1] Briefly, PEG113-Br (0.1 mmol), AA (3 mmol) and N, N, N', N'', N''-pentamethyldiethylenetriamine (PMDETA, 0. 1 mmol) were dissolved in 6 mL of anhydrous N, N-Dimethylacetamide (DMAC), degassed by three freezing-thawing cycle under vacuum condition, added with 14.4 mg of CuBr (0.1 mmol) under argon protection. The polymerization was conducted overnight at 40 °C. The reaction product was purified by passing through a neutral alumina column and dialyzing against DI water. The final product was obtained after lyophilization (yield 63%). ^{1}H NMR (500 MHz, CDCl3): δ 1.17 (s, 46H, -CCH2C-), 1.85 (s, 75H, -CCH3), 2.94 (s, 46H, -CH2CH2NH2), 3.31 (s, 3H, -OCH3), 3.58 (m, 454H, -OCH2CH2-), 4.39 (s, 46H, -OCH2CH2-). FTIR: 1744(C=O), 1650, 1549 cm^{-1}. The molecular weight of PEG-PAA-Br was calculated as 8000 with narrow PDI (1.16) by GPC. The ^{1}H NMR, FTIR and GPC spectrum of PEG-PAA-Br were shown in **Figure S1** b, **Figure S2** b, **Figure 1**B and **Table S1**, respectively.

Synthesis of PEG-P(AA-DA)-Br. The pH of the aqueous solution of mPEG113-PAA-Br was first adjusted to 8.5 using 1.0 M sodium hydroxide (NaOH) aqueous solution. Dimethylmaleic anhydride (DMMA) with twice the amount was gradually added to the solution, and the pH was also kept in the range of 8.0-9.0 using 1.0 M NaOH aqueous solution. When pH was stabilized, the reaction continued at room temperature for another 12 h. Then, the solution was dialyzed (MWCO = 3500 Da) against NaOH aqueous solution (pH 8-9) for another 12 h and lyophilized (yield 94%). ^{1}H NMR (500 MHz, CDCl3): δ 1.17 (s, 46H, -CCH2C-), 1.72-1.76 (m, 153H, -CCH3&-CCH3CCH3), 2.82 (s, 46H, -CH2CH2NH2&-CH2CH2NH-), 3.32 (s, 3H, -OCH3), 3.5 (m, 454H, -OCH2CH2-), 4.3 (s, 46H, -OCH2CH2-). FTIR: 1755 (C=O), 1683, 1550 cm^{-1}. The molecular weight of PEG-P(AA-DA)-Br was calculated as 10100 with narrow PDI (1.34) by

GPC. The ^{1}H NMR, FTIR and GPC spectrum of PEG-P(AA-DA)-Br were shown in **Figure S1** c, **Figure S2** c, **Figure 1**B and **Table S1**, respectively.

Synthesis of CPTMA prodrug. CPTMA prodrug polymer was synthesized through two-step reaction. Firstly, the hydroxyl group of 2-Hydroxyethyl methacrylate (HEMA) was activated by oxalic dichloride. Briefly, HEMA (10 mmol) in anhydrous DCM (8 mL) was dropwise added into oxalic dichloride (15 mmol) in anhydrous DCM (10 mL) at 0 °C, followed by stirring for 1 h. The excess oxalic dichloride and solvent were removed under reduced pressure to afford some colorless oil (yield 93%). Next, CPT (1.15 mmol) and triethylamine (1.8 mmol) were dissolved in anhydrous DCM (10 mL) at 0 °C, followed by addition of the prepared colorless oil (1.8 mmol) in anhydrous DCM (10 mL). After 1 h of reaction, the mixture was filtered and the solvent was evaporated under reduced pressure. Then the concentrated mixture was subjected to chromatographic separation (silica gel, DCM/ethyl acetate (4:1, v/v)) to yield CPTMA prodrug monomer as a pale-yellow solid (yield 71%). ESI-MS m/z (M+) calculate 533.1, found 533.47. ^{1}H NMR (300 MHz, δ, ppm, CDCl3): 8.41 (s, 1H), 8.22 (d, 1H), 7.96 (d, 1H), 7.85 (t, 1H),7.68 (t, 1H), 6.14 (s, 1H), 5.7 (d, 1H), 5.57 (s, 1H), 5.46 (d, 1H), 5.31 (s, 2H), 4.58(t, 2H), 4. 46 (t, 2H), 2.38-2.28 (m, 2H), 1.92 (s, 3H), 1.04 (t, 3H). FTIR: 1749, 1647, 1458, 1399, 1270, 1159 cm^{-1}. The ^{1}H NMR, FTIR and ESI-MS spectrum of CPTMA were shown in **Figure S1** d, **Figure S2** d and **Figure S1** f, respectively.

Synthesis of PEG-P(AA-DA)-CPTMA. PEG-P(AA-DA)-Br (0.01 mmol), CPTMA (0.52 mmol), and PMDETA (0.01 mmol) were dissolved in 8 mL of DMAC. The mixture solution was degassed by three freeze-pump-thaw cycles and stirred at room time for 10 min under nitrogen. CuBr (0.01 mol) was subsequently added into mixture solution under stirring. The reaction was continued at 40 °C for 16 h. The obtained reaction product was then diluted with THF and

purified a neutral alumina column to remove copper catalyst. The result product PEG-P(AA-DA)-CPTMA was collected by lyophilization (yield 98%). PEG113-P(PA10-DA13)-CPTMA16 was determined by ^{1}H NMR spectrum. The molecular weight of PEG-P(AA-DA)-CPTMA was calculated to be 16800 with a narrow PDI (1.29) by GPC and 17946 by ^{1}H NMR, respectively. ^{1}H NMR (400 MHz, CDCl3, ppm): 8.46 (m, 16H, -CH- in CPT chain), 8.28 (d, 16H, -CH- in CPT chain), 7.95 (dd, 16H, -CH- in CPT chain), 7.86 (dd, 16H, -CH- in CPT chain), 7.7 (m, 16H, -CH- in CPT chain), 5.41-5.35 (m, 64H, -CH2- in CPT chain), 4.48-4.2 (m, 78H, -OCH2CH2NH&-OCH2CH2O-), 3.44 (m, 454H, -OCH2CH2-), 3.17 (s, 3H, -OCH3), 2.67 (s, 54H, -CH2CH2NH2&-CH2CH2NH-), 2.36 (dd, 32H, -CH2- in CPT chain), 1.77-1.74 (m, 186H, -CCH3&-CCH3CCH3), 1.24 (s, 77H, -C-CH2CCH3). FTIR: 1750 (C=O), 1675, 1556, 1464, 1280, 1112 cm^{-1}. The ^{1}H NMR, FTIR and GPC spectrum of PEG-P(AA-DA)-CPTMA were shown in **Figure S1** e, **Figure S2** e, **Figure 1**B and **Table S1**, respectively.

Preparation of micelles and β-lapachone loaded micelles. The formation of micelles or β-lapachone loaded micelles were achieved by an evaporation method as reported previously.[2] 200 mg of the polymer prodrug combined with/without 20 mg β-lapachone were dissolved in 4 mL DCM. After stirring for 4 h, the mixture solution was added to deionized water (16 mL) using infusion pump at a constant rate of 2 mL/h. After stirring at room temperature for 24 h, the dispersion was dialyzed against deionized water (MWCO 3.4 kDa) for 48 h and freeze-dried. The resulting polymer was then dissolved with deionized water and filtered with Millex® GP Filter Unit (0.45 μm, Millipore). The obtained micelles were denoted as PPDC and PPDC@β-lapachone, respectively.

The mass fraction of CPT in PPDC was quantified by UV/Vis spectroscopy based on the standard curve of CPT at λ = 365 nm. The content of β-lapachone in PPDC@β-lapachone was

measured using UV-Vis spectroscopy as well (λ = 268 nm). Drug loading content (DLC) and drug loading efficiency (DLE) were calculated according to our previous study and listed as follow: [2]

DLC = Amount of loaded drug / weight of copolymer × 100 %

DLE = Amount of loaded drug / weight of drug in feed × 100 %

Materials Characterization. Molecular weights and polydispersity index of the copolymers were measured by a gel-permeation chromatograph (GPC) equipped with PL gel, 5 mm MIXED-C columns (Waters 1424 detector, Agilent Technologies, USA). The eluent used was THF, and the flow rate was 1.0 mL min-1 at 40 °C. 1H NMR (Bruker Avance 500 MHz, Swiss), mass spectrometry (Waters Acquity SQ Detector UPLC-MS) and Fourier transforms infrared spectroscopy (FTIR, model 6300, BioRad Co. Ltd., USA) were employed to analyze the structure and weight of the copolymers. The morphology of the micelle was characterized by transmission electron microscopy (TEM, LIBRA 200 CS, Carl Zeiss Co., Germany). The size distribution of micelles in aqueous solution were measured by ζ-potential measurement (Nano ZS90 Zetasizer, Malvern Instruments Co. Ltd., U.K.) equipped with DLS. HPLC was performed on a C18 column (Bioband HP-120, 4.6 mm × 150 mm, 5 µm) using a linear gradient of acetonitrile and DI water containing 0.1% TFA as a mobile phase with the flow rate of 0.6 mL/min at 25 °C.

Critical micelle concentration (CMC). The CMC of PPDC micelles were measured by fluorescence spectrometry according to our previous study. [3]

Drug release behavior. PPDC@β-lapachone micelles (3 mg, abbreviated as PPDC@β-Lap) suspended in 1 mL of PBS (pH 7.4) containing various concentrations of H_2O_2 (0, 0.1, and 10

mM) were sealed in a dialysis bag (MWCO 3.4 kDa). The dialysis tubes were subsequently immersed into glass tube containing 29 mL of PBS (pH 7.4) with same concentrations of H_2O_2 and 2% Tween 80. At predetermined time intervals, a 300 μL aliquot of the solution outside the bag was taken out for HPLC analysis and replaced with the same volume of fresh medium.

Protein adsorption. Bovine serum albumin (BSA) was used as a model protein to examine protein adsorption capacity of PPDC micelles according to a previous study. [4] PPDC (1 mg/mL) were incubated with 2 mg/mL of BSA in pH 7.4 and pH 6.0 PBS at 37 °C for 12 h, respectively. Then each sample was centrifuged to precipitate the protein-adsorbed micelles. The concentration of residual BSA in PBS solution was measured using uv-vis spectroscopy (Lambda 900, PerkinElmer instruments, USA), and the amount of BSA adsorbed on the micelles was then obtained.

Measurement of intracellular ROS generation. DCF-DA was used to monitor ROS generation in MCF-7 cells. Typically, MCF-7 cells seeded on 6-well plates or confocal microscopy dishes were treated with 2 μM β-lapachone, and PPDC@β-Lap +/- dicoumarol (60 μM) for 2 h and then incubated with fresh medium containing DCF-DA for 20 min. Next, the cells seeded on confocal microscopy dishes were washed with fresh DMEM and imaged by CLSM. Additionally, the cells seeded on 6-well plates were harvested using 0.25% (w/v) trypsin/0.02% (w/v) EDTA and then suspended into cell binding solution. The final cell samples were quantitatively analyzed by FCM (BD Biosciences) in the FL1 channel.

Cell culture and cell viability assay. Human breast carcinoma cells (MCF-7), MCF-7 drug resistant cells (MCF-7 ADR), murine embryo fibroblast NIH/3T3 cells were purchased from Cell Bank of Chinese Academy of Sciences (Shanghai, China). These cells were grown in

Dulbecco's modified Eagle's medium (DMEM) supplemented with 10 % (v/v) fetal bovine serum (FBS, Gibco) and 1 % (w/v) penicillin (100 U/mL)/streptomycin (100 μg/mL) with 5% CO_2 at 37 °C.

For cell viability assay, MCF-7 cells seeded on 24-well plate (2×10^4 cells per cm^2) were treated with PBS (Control), CPT (5.1 μM), β-lapchone (5 μM), PPDC (7.2 μg/mL, equivalent of 5.1 μM CPT) and PPDC@β-Lap (7.2 μg/mL, the same dosage as those of CPT and β-lapachone) with or without the NQO1 inhibitor dicoumarol (60 μM) for 48 h, and then the medium was replaced by mixture solution containing 200 μL fresh medium and 20 μL CCK-8. After 1.5 h of incubation at 37 °C, the absorbance in each well was recorded with a spectrophotometric microplate reader (Bio-Rad 680, USA) at 450 nm.

Moreover, the cytotoxicity of PPDC@β-Lap against normal NIH/3T3 cells was evaluated as above. Briefly, NIH/3T3 cells were incubated with PPDC@β-Lap at various concentrations (equivalent β-lapachone concentration ranged from 0 to 5 μM) for 48 h. Cell viability were calculated by CCK8 assay as described above.

***In vitro* cellular uptake, distribution and intracellular drug release.** The pH-enhanced cell uptake of PPDC@β-Lap micelles in MCF-7 cells was visualized by CLSM and quantitatively analyzed by FCM. MCF-7 cells seeding on confocal microscopy dishes/6-well plates (10^5 cells per well) were incubated in DMEM at pH 7.4 and 6.8 containing PPDC@β-Lap without or with NQO1 inhibitor dicoumarol for 12 h. Cells were then fixed with 4% paraformaldehyde, permeabilized with 0.5 % TritonX-100, stained with Alexa 488-phalloidin and Reddot-2, following by observed with CLSM (LSM 510 META Olympus, Japan). For FCM analysis, all cells were then washed with PBS, collected by centrifugation (2000 rpm×10 min, 4 °C), and re-

suspended in cell binding solution. Cellular uptake and intracellular CPT release were analyzed by FCM (BD, Biosciences).

Measurement of intracellular ATP level. CellTiter-Glo®luminescent cell viability assay kit (Promega, Inc., Madison, WI) was employed to measure the intracellular ATP concentration. Typically, MCF-7 ADR cells were incubated with 5 μM β-lapachone, 3.6 μM CPT or the equivalent dose of PPDC or PPDC@β-Lap with or without dicoumarol (60 μM) in 96-well plates for 4 h. Then, the culture solution was replaced with 100 μL of fresh medium and 100 μL of CellTiter-Glo reagent for each well, and the cell samples were further shaken for 2 min and incubated at room temperature for 10 min. The mixture solution was measured a Synergy™ HTX multi-mode microplate reader (Biotek, America). The untreated cells were taken as control group.

FCM analysis of cells apoptosis. MCF-7 ADR cultured on 6-well plates were treated with PBS (Control), CPT (5.1 μM), β-lapachone (5 μM), PPDC (7.2 μg/mL, equivalent of 5.1 μM CPT) and PPDC@β-Lap (7.2 μg/mL, the same dosage as those of CPT and β-lapachone) with or without the NQO1 inhibitor dicoumarol (60 μM) for 24 h. Cells were collected via centrifugation and then analyzed in the FL1/FL3 channel on FCM using a Annexin V-FITC/PI kit (NeoBioscience, China) staining kit.

Western-blot analysis. MCF-7 ADR cells were seeded on 6-well plates and and allowed for a cell confluence of 60-70%. Afterward, cells were divided to 6 group and respective treated with different treatments as described above for 48 h. Subsequently, cells were solubilized with lysis buffer and collected by centrifugation (12000 rpm × 10 min, 4 °C). BCA protein assay kit (Beyotime) was used to determine the protein concentration. The protein samples were analyzed for P-gp using Western blotting and Image J software 1.45f. The detailed

experimental protocols of western-blot assay were performed according to previous literatures. [2, 3]

***In vivo* antitumor efficacy.** Male nude mice (5-6 weeks old) were purchased from Beijing Institution for Drug Control, China. MCF-7 cell tumor-bearing mice model was established by subcutaneous injection of MCF-7 cells (1×10^6) into the right groin side of each mouse. Notably, all animal experiments were strictly performed according to guidelines of the Institutional Animal Care and Use Committee of China. After tumor volume reached 50-100 mm^3, tumor-bearing mice were randomly divided to five groups and intravenously injected with saline, CPT, β-lapachone, PPDC and PPDC@β-Lap micelles at the dose of 3 mg/kg CPT equivalent (n= 6). Above administrations were given 2 times per week and continued for 20 days. The body weights and tumor volumes of tumor-bearing mice were recorded per 2 days. Tumor volume (V) was calculated according to following formula: V = the longest dimension × the shortest dimension^2/2. The survival rate of mice was monitored for another 20 days after the last administration.

H&E, TUNEL, and immunofluorescence (IFC) assays. The mice were sacrificed at the end of antitumor studies, the major organs (liver, heart, kidney, spleen and lung) and tumor were harvested, fixed, embedded and sliced for H&E examinations; Tumor sections were also stained with TUNEL apoptosis detection kit (Beyotime) and then observed by CLSM; For IF assays, tumor sections were deparaffinized, hydrated, blocked with 5% BSA, incubated with Ki67 antibody, stained with Alexa Fluor 488-labeled goat anti-mouse IgG (Immunol Fluorence Staining Kit, Beyotime, China) and DAPI, following by observed with CLSM. The detailed staining protocols of western- H&E, TUNEL and IFC were performed according to our previous literature. [5]

Pharmacokinetics and biodistribution study. In the plasma pharmacokinetic study, MCF-7 cell tumor-bearing nude mice were intravenously administered with CPT or PPDC@β-Lap micelles at a dose of 3 mg/kg CPT. At predetermined time points (0, 1, 2, 4, 8, 12 and 24 h), blood samples were collected into heparinized tubes and then centrifuged to obtain plasma. Subsequently, acetonitrile was added to above solution for protein settlement, followed by centrifugation at 12000 rpm for 8 min to collect the supernatant. Finally, the collected sample were dried, re-dissolved and detected by HPLC to determine the CPT levels.

To assess the distribution of micelle formulation on tissues, MCF-7 cell tumor-bearing nude mice were injected intravenously with CPT and PPDC@β-Lap prodrug micelles at a dose of 3 mg/kg CPT. The mice were sacrificed after 24 h post-injection. Then the major tissue (heart, liver, spleen, lung, kidney and tumor) were excised, washed, cut into small pieces, and homogenized in 0.5 mL of DMSO, followed by centrifugation at 15000 rpm for 15 min. The amounts of CPT inside each tissue were measured by fluorescence spectrophotometry according to previous literature. [2] The distribution of micelle in tissues was expressed as μg (amount of micelle)/g (weight of tissues).

Statistical Analysis. We performed the statistical analysis with software of OriginPro (version 9.0) through Student's t-test and one-way analysis of variance (ANOVA). All data were expressed as means ± standard deviation (SD). The confidence levels of 95 % and 99 % were regarded as significant difference.

a

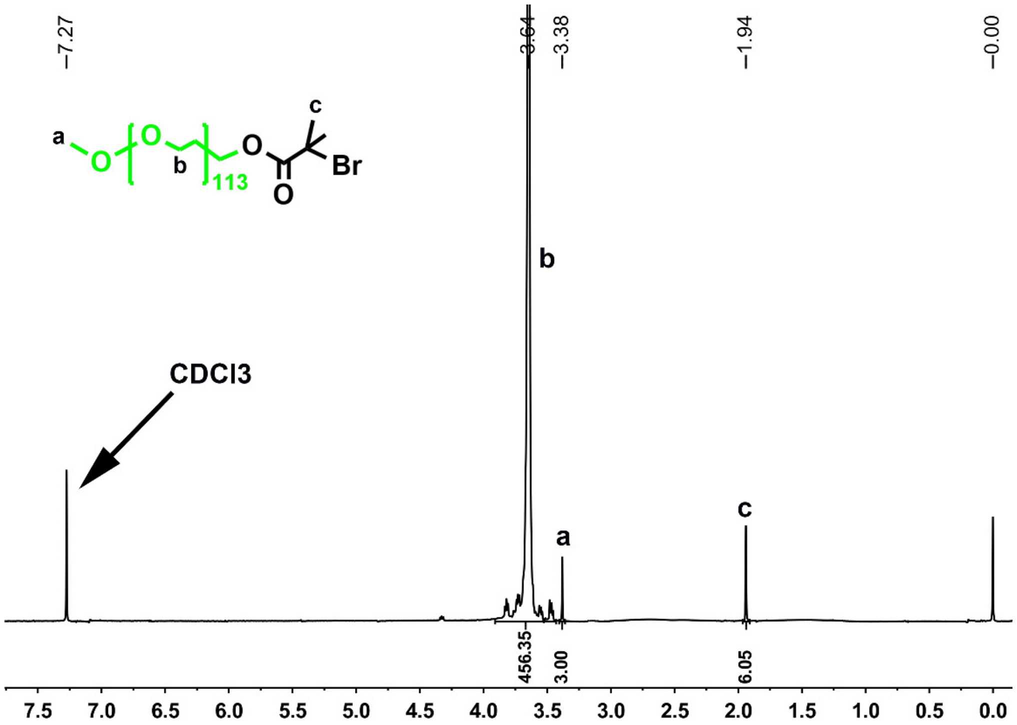
-7.27
3.64
-3.38
-1.94
-0.00
a
b
113
c
Br
O
CDCl3
b
a
c
456.35
3.00
6.05
7.5 7.0 6.5 6.0 5.5 5.0 4.5 4.0 3.5 3.0 2.5 2.0 1.5 1.0 0.5 0.0

b

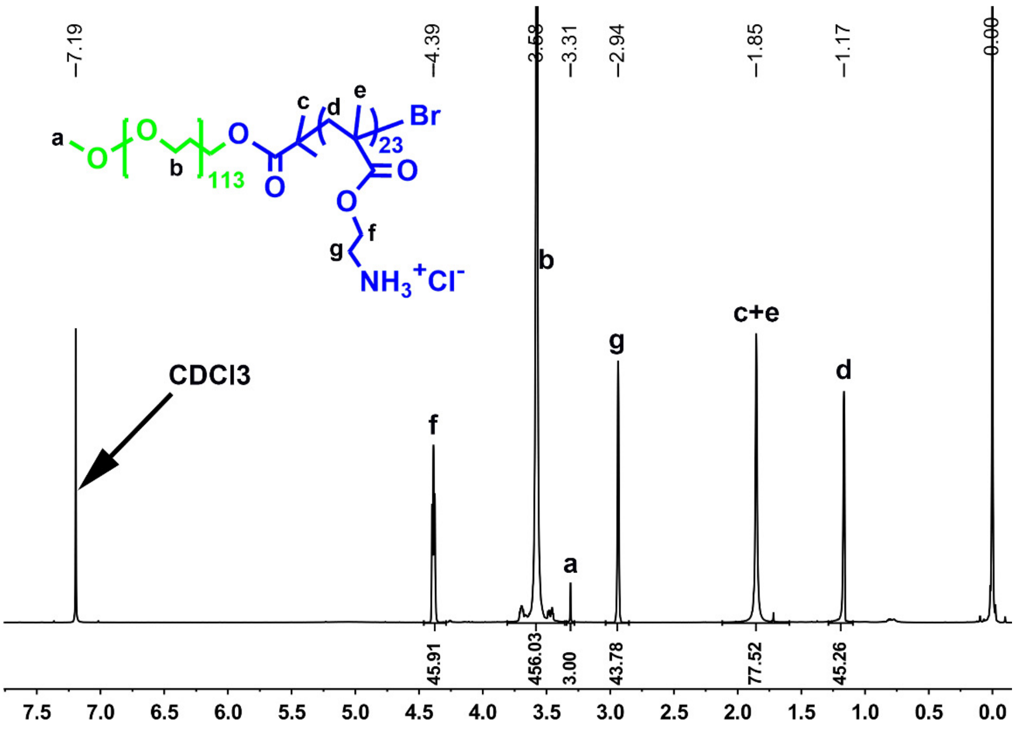
-7.19
-4.39
3.58
-3.31
-2.94
-1.85
-1.17
0.00
a
b
113
c
d
e
23
Br
O
f
g
$NH_3^+Cl^-$
CDCl3
f
b
a
g
c+e
d
45.91
456.03
3.00
43.78
77.52
45.26
7.5 7.0 6.5 6.0 5.5 5.0 4.5 4.0 3.5 3.0 2.5 2.0 1.5 1.0 0.5 0.0

c

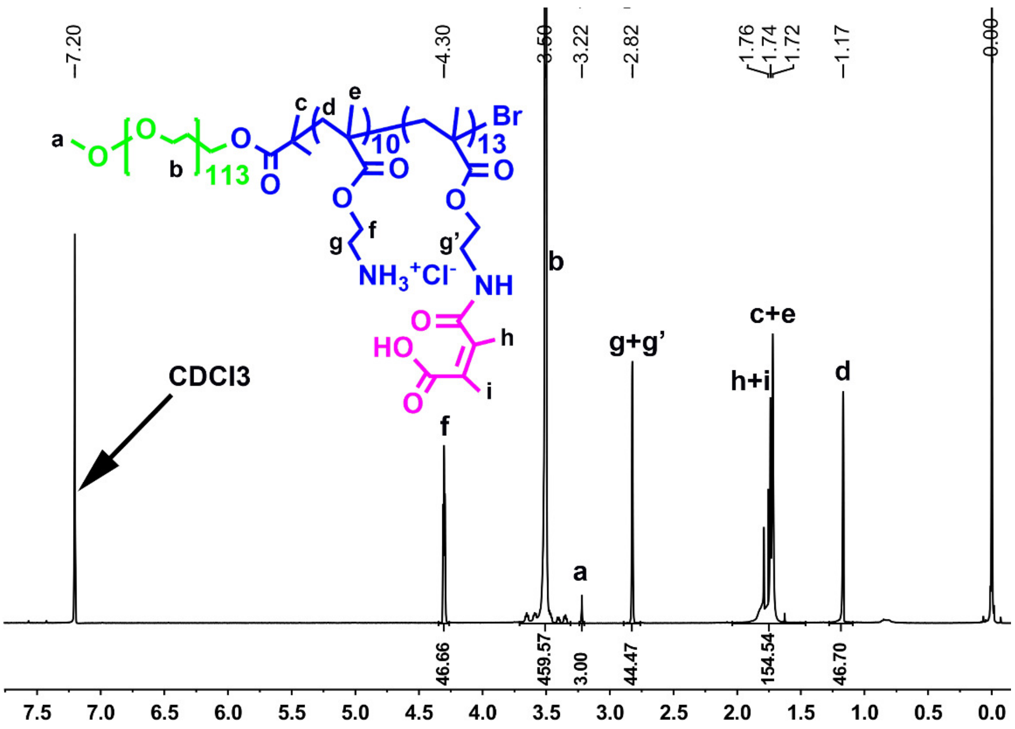
CDCl3
a
b
113
10
13
Br
c
d
e
f
g
g'
$NH_3^+Cl^-$
NH
HO
h
i
g+g'
c+e
h+i
d
7.20
4.30
3.50
3.22
2.82
1.76
1.74
1.72
1.17
0.00
46.66
459.57
3.00
44.47
154.54
46.70
7.5 7.0 6.5 6.0 5.5 5.0 4.5 4.0 3.5 3.0 2.5 2.0 1.5 1.0 0.5 0.0

d

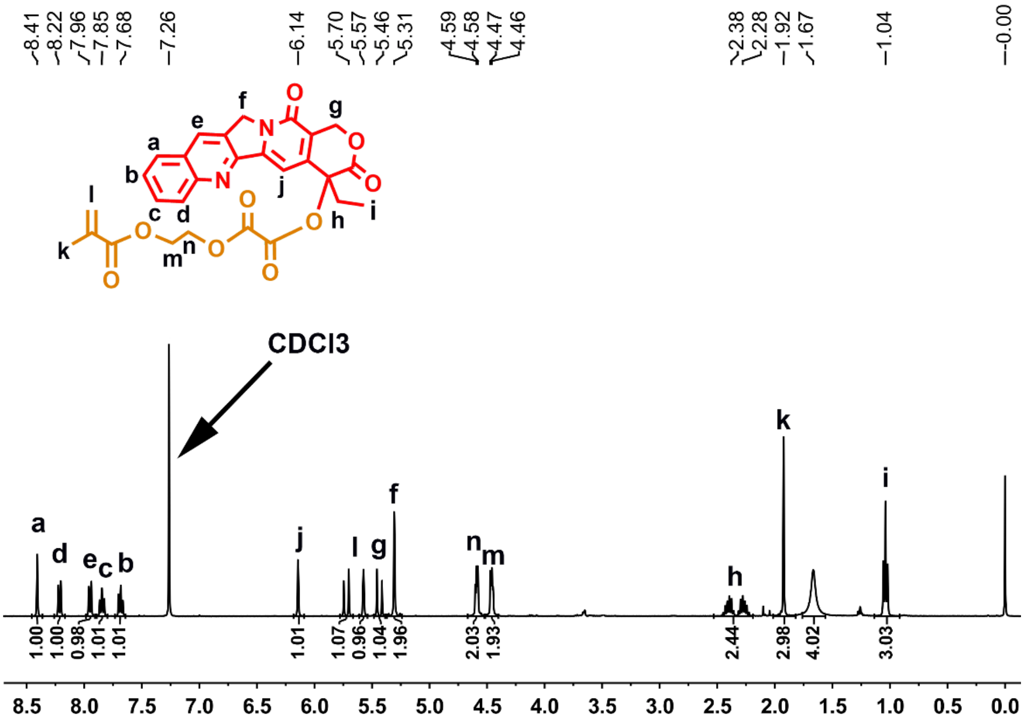
CDCl3
a
b
c
d
e
f
g
h
i
j
k
l
m
n
8.41
8.22
7.96
7.85
7.68
7.26
6.14
5.70
5.57
5.46
5.31
4.59
4.58
4.47
4.46
2.38
2.28
1.92
1.67
1.04
0.00
1.00
1.00
0.98
1.01
1.01
1.01
1.07
0.96
1.04
1.96
2.03
1.93
2.44
2.98
4.02
3.03
8.5 8.0 7.5 7.0 6.5 6.0 5.5 5.0 4.5 4.0 3.5 3.0 2.5 2.0 1.5 1.0 0.5 0.0

e

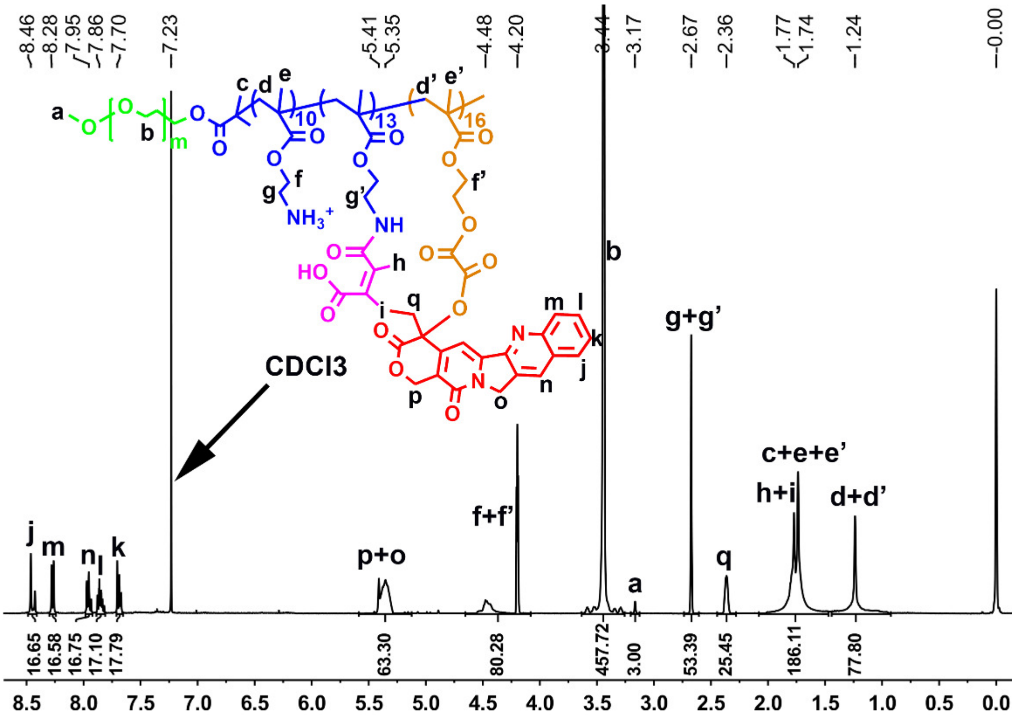

f

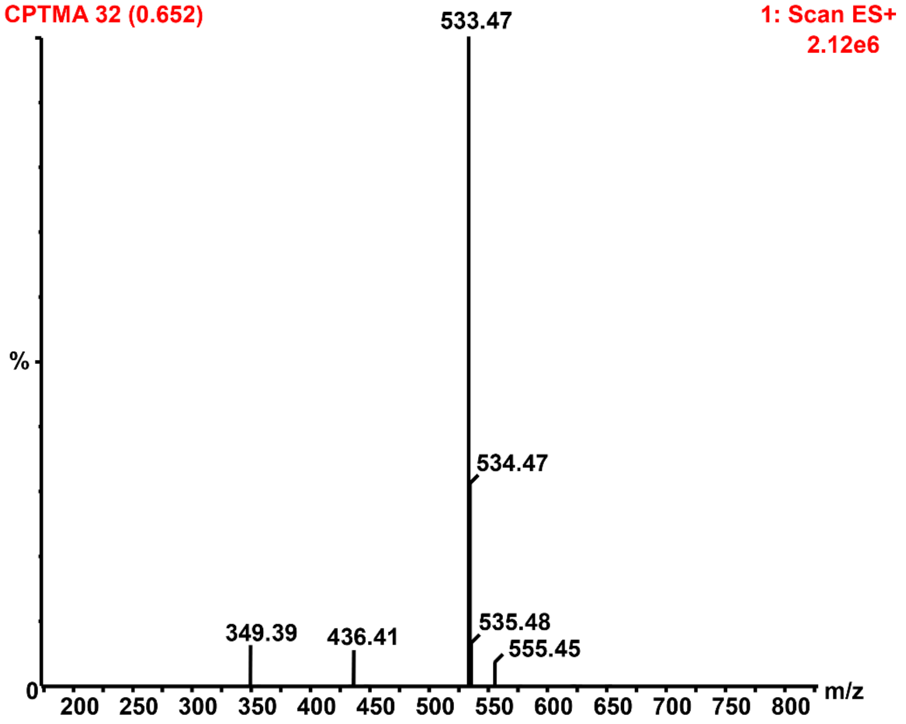

Figure S1. Characterization of PEG-P(AA-DA)-CPTMA prodrug copolymer: 1H NMR spectra

(400 MHz, 298 K) of relevant products from the synthesis process of PEG-P(AA-DA)-CPTMA copolymer, including PEG-Br (a), PEG-PAA-Br (b), PEG-P(AA-DA)-Br (c), CPT-MA (d) and PEG-P(AA-DA)-CPTMA (e); and ESI-MS spectrum of CPT-MA (f).

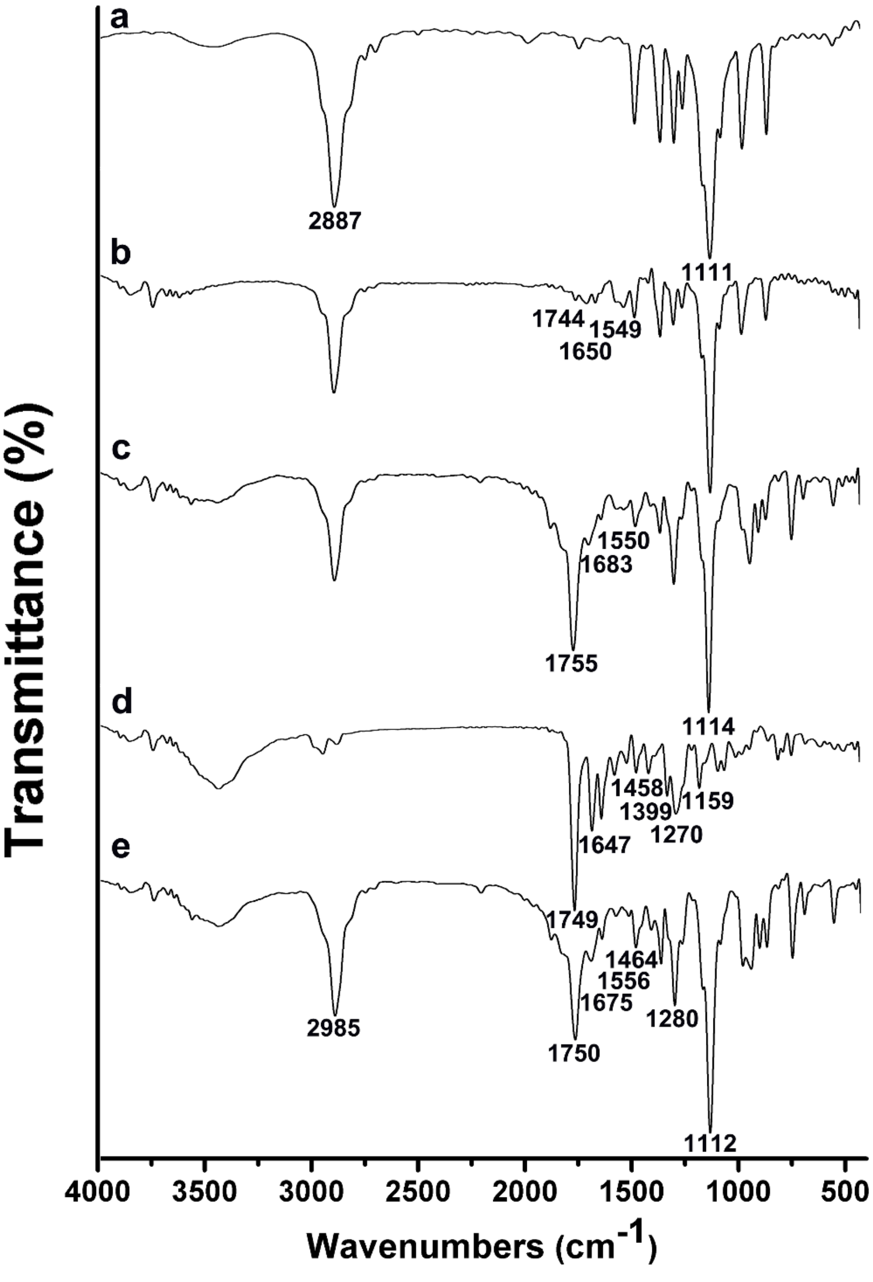

Figure S2. FTIR spectra of (a) PEG-Br, (b) PEG-PAA-Br, (c) PEG-P(AA-DA)-Br, (d) CPTMA, and (e) PEG-P(AA-DA)-CPTMA, respectively.

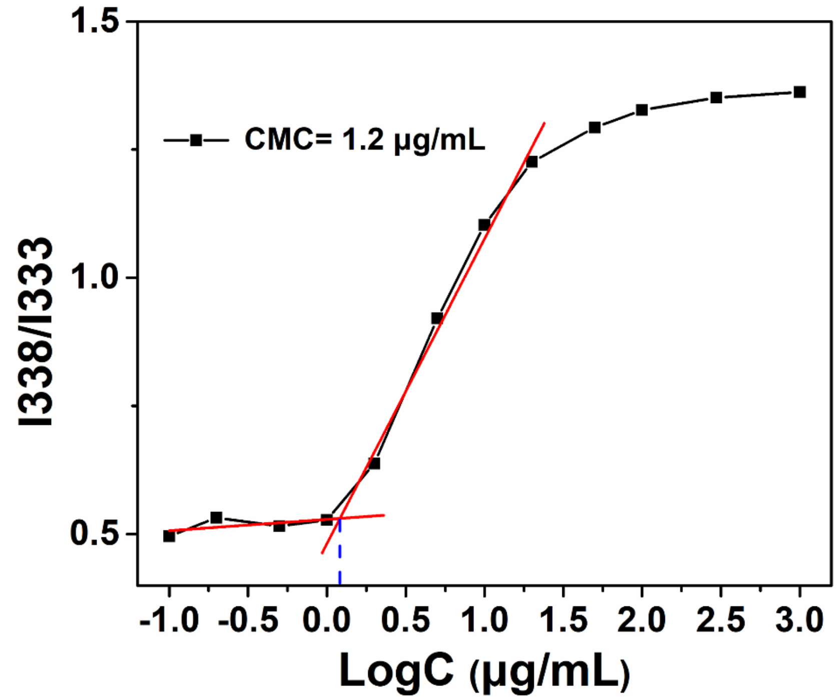

Figure S3. Critical micelle concentration (CMC) of PEG-P(AA-DA)-CPTMA micelle.

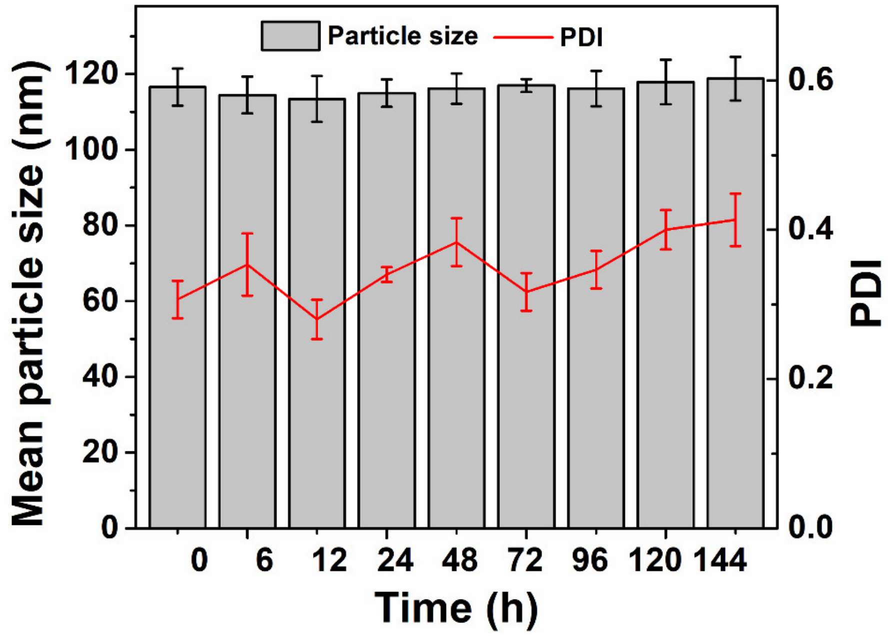

Figure S4. Stability of PPDC@β-Lap micelles incubated in serum for 6 days.

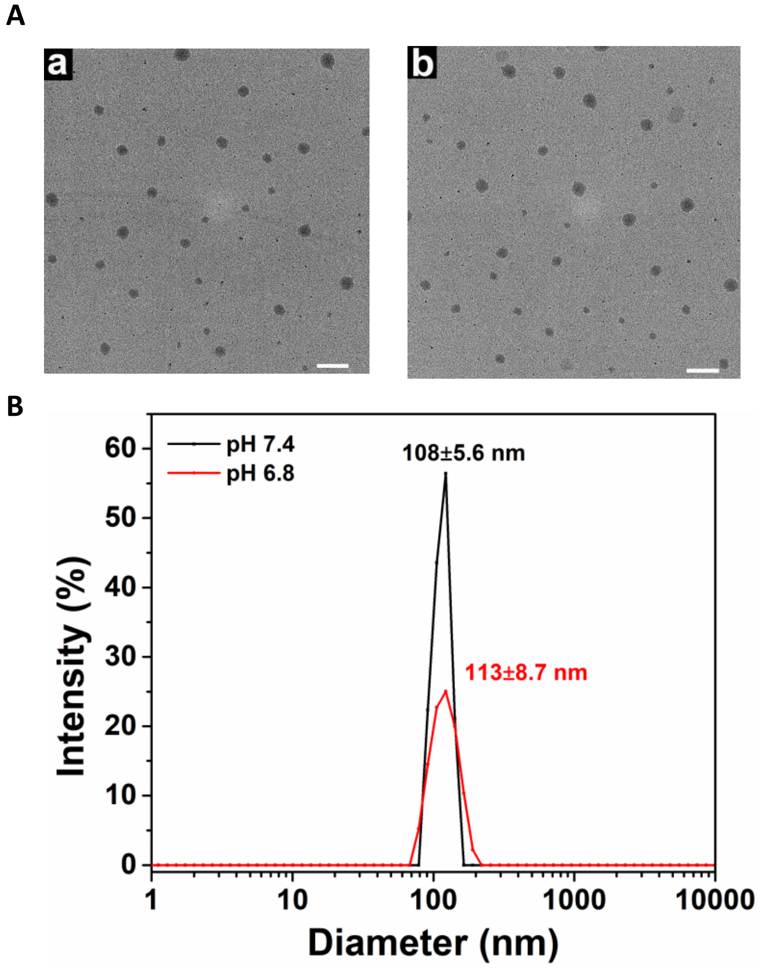

Figure S5. (A) TEM images and (B) DLS analysis of PPDC@β-Lap micelles in pH 7.4 (a) and 6.8 (b). Scale bars: 100 nm

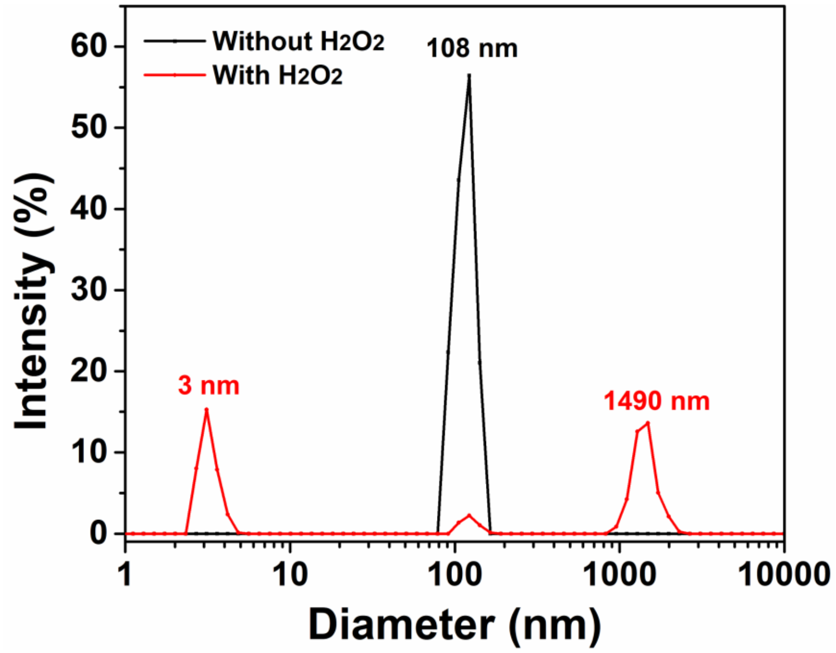

Figure S6. DLS size distribution of the PPDC@β-Lap dissolved in pH 7.4 PBS solution in the absence and presence of H_2O_2 (1 mM) for 2 h, respectively.

A

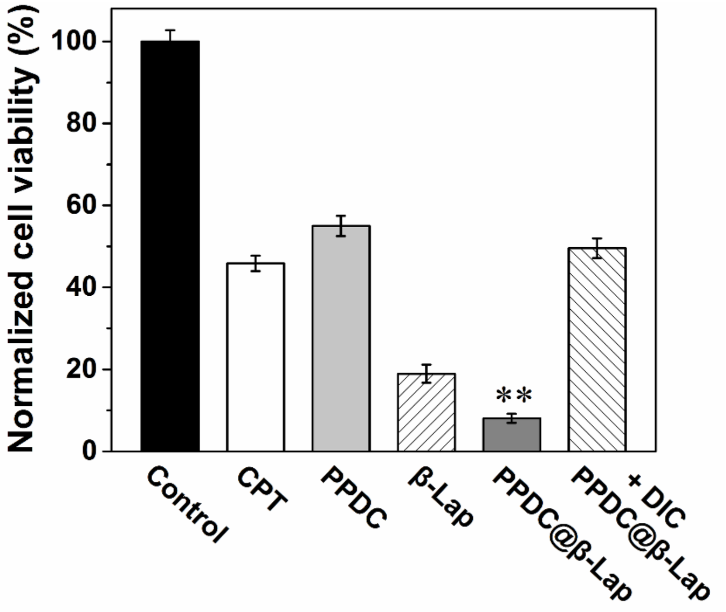

B

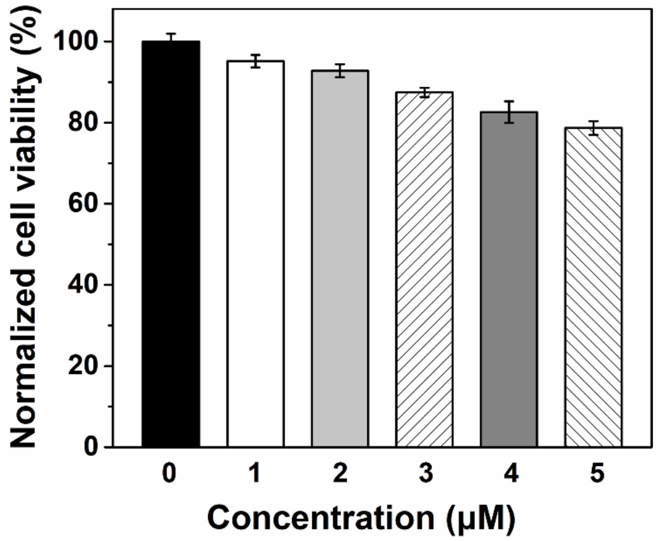

Figure S7. (A) Cytotoxicity assays of CPT (5.1 μM), β-lapachone (5 μM), PPDC (7.2 μg/mL) and PPDC@β-Lap with or without the NQO1 inhibitor dicoumarol (60 μM) over 48 h in MCF-7 breast cancer cells; (B) Concentrations-dependent cell viability of NIH/3T3 cells treated with various concentrations of PPDC@β-Lap micelles (equivalent β-lapachone concentration ranged from 0 to 5 μM) for 48 h, respectively. Error bars present as mean ± SD (n=4), **p < 0.01.

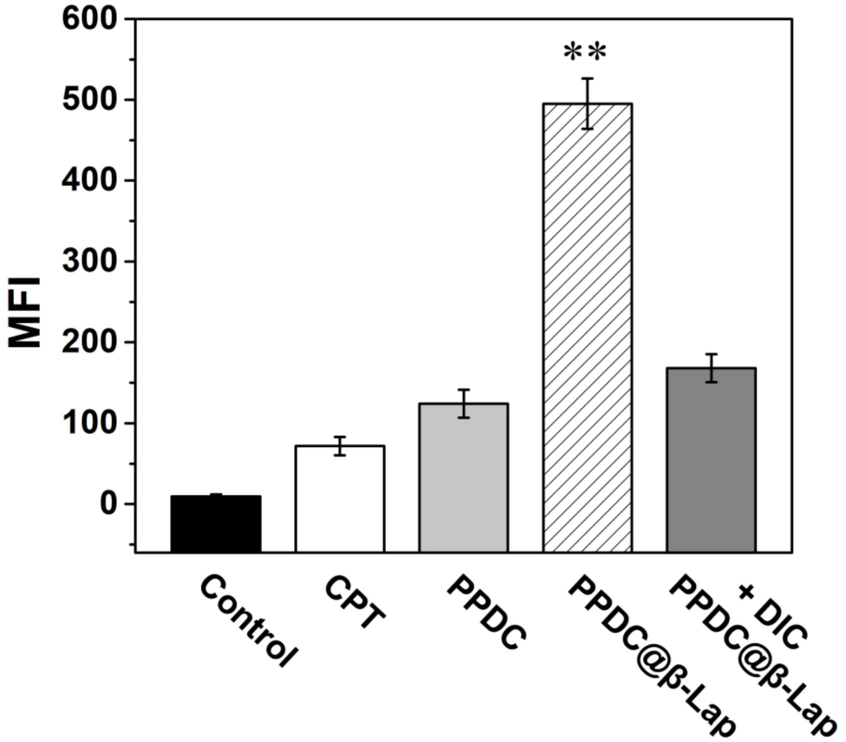

Figure S8. Mean fluorescence intensity of CPT fluorescence in MCF-7 ADR cells after incubation with CPT, PPDC, and PPDC@β-Lap with or without the NQO1 inhibitor dicoumarol for 12 h, respectively. Error bars represent means ± SD (n=4), **$p < 0.01$.

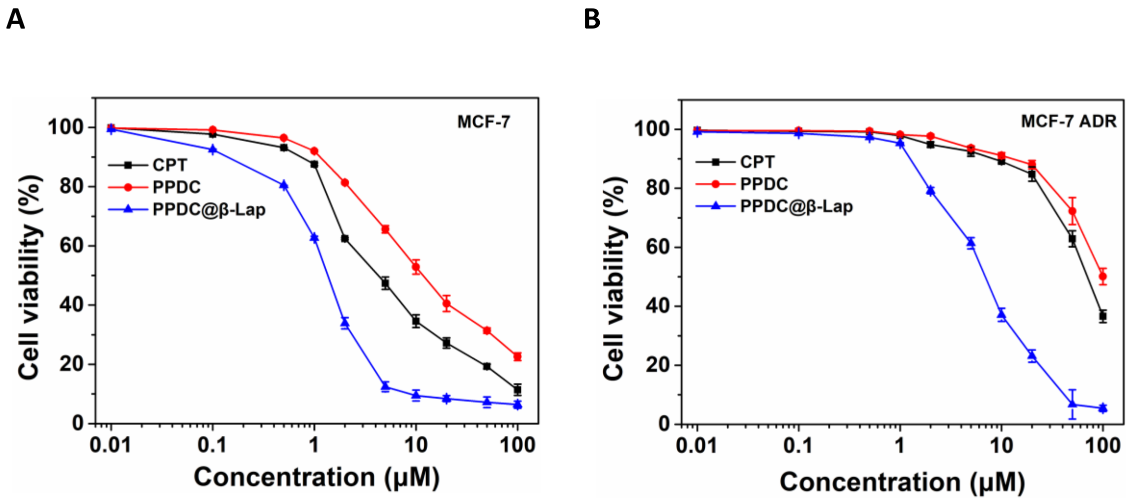

Figure S9. Cytotoxicity assays of CPT, PPDC and PPDC@β-Lap over 48 h in MCF-7 cells (A) and MCF-7 ADR multidrug-resistant cells (B).

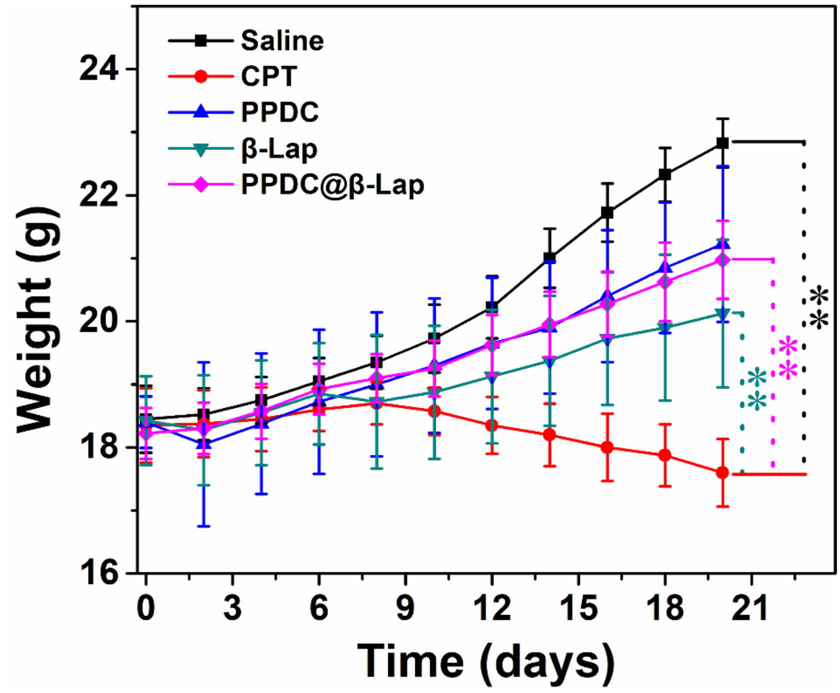

Figure S10. The curve of weight change of mice bearing MCF-7 tumors after different treatments. Error bars present as mean ± SD (n=6), $**p < 0.01$.

Table S1. Molecular weight data of various intermediate products in the synthesis process of PEG-P(AA-DA)-CPTMA copolymer.

Polymer	$Mn\text{-}_{theory}$	$Mn\text{-}_{NMR}$	$Mn\text{-}_{GPC}$	PDI
PEG-PAA-Br	8094	8033	8000	1.16
PEG-P(AA-DA)-Br	9732	9671	9500	1.32
PEG-P(AA-DA)-CPTMA	18244	17946	16800	1.29

Mn-theory: Theoretical molecular weight calculation based on monomer conversion.

$Mn\text{-}_{NMR}$: Molecular weight based on ^{1}HNMR analysis.

$Mn\text{-}_{GPC}$: Number-average absolute molecular weight determined by GPC equipped with refractive index and multi-angle light scattering detectors.

PDI: Polydispersity index determined by GPC analysis.

Table S2. IC50 and CI of CPT and β-lapachone in different formulations against MCF-7 ADR cells.

	IC50 (μg/mL)			
Formulations	CPT	β-lapachone	PPDC@β-Lap	CI
CPR	25.85	/	/	/
β-lapachone	/	0.71	/	/
PPDC@β-Lap	0.62	0.27	2.52	0.44

The combination index (CI) was calculated using the following formula:

$$CI = C1 / C_C + C2 / C_L$$

C1: IC50 value of CPT in the PPDCβ-Lap.

C2: IC50 value of β-lapachone in the PPDCβ-Lap.

C_C: IC50 value of free CPT.

C_L: IC50 value of free β-lapachone.

References

[1] P. D. Topham, N. Sandon, Read, E. S. Read, J. Madsen, A. J. Ryan, S. P. Armes, *Macromolecules*. **2008**, *41*, 9542.

[2] L. L. Dai, R. S. Cai, M. H. Li, Z. Luo, Y. L. Yu, W. Z. Chen, X. K. Shen, Y. X. Pei, X. J. Zhao, K. Y. Cai, *Chem. Mater.* **2017**, *29*, 6976.

[3] L. L. Dai, Y. L. Yu, Z. Luo, M. H. Li, W. Z. Chen, X. K. Shen, F. Chen, Q. Sun, Q. F. Zhang, H. Gu, K. Y. Cai, *Biomaterials* **2016**, *104*, 1.

[4] H. Z. Deng, J. J. Liu, X. F. Zhao, Y. M. Zhang, J. F. Liu, S. X. Xu, L. D. Deng, A. J. Dong, J. H. Zhang, *Biomacromolecules*. **2014**, 1*5*, 4281-4292.

[5] L. L. Dai, K. Li, M. H. Li, X. J. Zhao, Z. Luo, L. Lu, Y. F. Luo, K. Y. Cai, *Adv. Funct. Mater.* **2018**, *28*, 1707249.
